# Reducing complexity and unidentifiability when modelling human atrial cells

**DOI:** 10.1098/rsta.2019.0339

**Published:** 2020-05-25

**Authors:** C. Houston, B. Marchand, L. Engelbert, C. D. Cantwell

**Affiliations:** 1 ElectroCardioMaths Programme, Centre for Cardiac Engineering, Imperial College, London, UK; 2 Department of Aeronautics, Imperial College, London, UK

**Keywords:** cardiac modelling, approximate Bayesian computation, uncertainty, unidentifiability, action potential

## Abstract

Mathematical models of a cellular action potential (AP) in cardiac modelling have become increasingly complex, particularly in gating kinetics, which control the opening and closing of individual ion channel currents. As cardiac models advance towards use in personalized medicine to inform clinical decision-making, it is critical to understand the uncertainty hidden in parameter estimates from their calibration to experimental data. This study applies approximate Bayesian computation to re-calibrate the gating kinetics of four ion channels in two existing human atrial cell models to their original datasets, providing a measure of uncertainty and indication of potential issues with selecting a single unique value given the available experimental data. Two approaches are investigated to reduce the uncertainty present: re-calibrating the models to a more complete dataset and using a less complex formulation with fewer parameters to constrain. The re-calibrated models are inserted back into the full cell model to study the overall effect on the AP. The use of more complete datasets does not eliminate uncertainty present in parameter estimates. The less complex model, particularly for the fast sodium current, gave a better fit to experimental data alongside lower parameter uncertainty and improved computational speed.

This article is part of the theme issue ‘Uncertainty quantification in cardiac and cardiovascular modelling and simulation’.

## 1. Introduction

A central component in simulations of cardiac electrophysiology is a model of an action potential (AP) for a representative cardiomyocyte. These models describe how the transmembrane potential, and other physiological properties of a cardiac cell, vary through time due to changing environmental conditions or applied stimuli. Since the development of the initial, relatively simple, model of a neuron [1], AP models have grown in scope and complexity as new experimental data have become available [2].

Uncertainty is an unavoidable aspect of scientific experiments, particularly in the biological sciences. Further understanding in this area has been designated a priority as cardiac models advance towards safety-critical applications [3]. Uncertainty is introduced by variability inherent in the biological system (e.g. differences in AP waveforms between different myocytes), the stochastic nature of biological processes (e.g. opening and closing of ion channels) and imperfect recording systems (e.g. noise in voltage patch-clamp experiments) [3]. It is common for AP models to use equations with a large number of parameters to describe the flow of ions across the cell membrane. This is a particular challenge for model calibration in which parameters are tuned to reflect experimental observations, usually by comparing model output to experimental measurements using a regression method (e.g. least squares). The consequence of the uncertainty and high number of parameters is that multiple different parameter combinations may produce the same residual to experimental data; a unique optimum parameter set may not exist.

Traditional fitting techniques, such as simple least-squares regression, implicitly assume that a single point estimate exists for each parameter in a model [4]. In cardiac modelling, this is unlikely to be the case for reasons outlined above. Bayesian methods can quantify uncertainties in parameter estimates by determining a posterior distribution over parameter values given the available data [5]. These distributions can highlight unidentifiable parameters, those without a single unique optimum, and make it possible to capture the effect of experimental and biological variability on model parameters. Unidentifiability may be either structural, caused by an overly complex model where parameters can be varied simulataneously without a change in model output, or practical, where insufficient data are available to determine a parameter’s value. In cardiac cell modelling, it can be difficult to obtain an exact likelihood function necessary for exact Bayesian inference when summary statistics are used, as is commonplace in studies of cardiac ion channels. This is further complicated by the high-dimensional parameter space, nonlinear nature and indirect observation properties of AP models [6]. Instead, one can employ approximate Bayesian methods, such as approximate Bayesian computation (ABC), which provide a reasonable estimate to the posterior distributions of parameter values [7].

When developing a model of a new cell type, the most common approach is to ‘inherit’ gating kinetic formulations from existing models and tune channel conductances to data from multiple sources [8]. However, equations describing gating kinetics of ion channels can be extremely complex and often contribute the majority of the parameters in AP models. In contrast to channel conductances, which can be adjusted to measurements of APs, gating kinetics are typically calibrated to data from voltage patch-clamp experiments. In these experiments, channels are isolated, by pharmacological means or the use of specific voltage step protocols, to take measurements of individual current traces. Previous studies [9–11] have been valuable in developing approaches to investigate parameter identifiability in both generalized Hodgkin–Huxley models and more detailed widely used channel models. We build on this field of work to include consideration of the available experimental data across a range of simple and complex channel models of a human atrial cell.

In this study, we apply an approximate Bayesian method to investigate the uncertainty and parameter unidentifiability present in channel gating kinetics in a human atrial cardiomyocyte. Computational experiments are carried out on two human atrial cell models, the Nygren [12] and Courtemanche [13] models, henceforth referred to as the N and C models, respectively. These were the first two biophysical models developed to simulate the AP from a human atrial myocyte and have proved influential in the development of subsequent models and whole-heart tissue-scale modelling. The N and C models are detailed cell models, each including descriptions of 12 ion currents which contribute to the AP in human atrial myocytes. We thus only focus our study on the four major ion currents which are prominent determinants of their AP morphology [14,15]: the fast sodium channel (*I*_Na_), L-type calcium channel (*I*_CaL_), transient outward potassium channel (*I*_to_) and ultra-rapid delayed rectifier potassium channel (*I*_Kur_).

We first re-calibrate parameters in each channel model to experimental datasets used in the original publications to investigate the existing level of uncertainty and parameter unidentifiability. To explore whether these issues can be alleviated from inclusion of more data, which would suggest practical unidentifiability, a ‘unified’ dataset is formed and the models re-calibrated to these data. To investigate whether these models suffer from structural unidentifiability, a model of reduced complexity [16] is calibrated to the same unified dataset, and parameter posterior distributions and the overall goodness-of-fit of the model compared to the re-calibrated N and C models. These re-calibrated channel models are then inserted into the full N and C cell models to study the effect of the re-calibration on AP morphology. We conclude by discussing the relative advantages and drawbacks of these approaches and limitations of the study.

## 2. Methods

### (a) Action potential models

The AP models studied in this work follow the commonly used Hodgkin–Huxley gating form [1]. The changing transmembrane voltage is calculated from the solution of several coupled ordinary differential equations describing individual ion currents. Each current is of the common form

2.1 $$I_{i}=g_{i}\prod_{j}\gamma_{j}^{k_{j}}f(V_{m})\;\text{and}\;\frac{\text{d}\gamma_{j}(t)}{\text{d}t}=\alpha_{\gamma_{j}}(V_{m};\lambda )[1-\gamma_{j}]-\beta_{\gamma_{j}}(V_{m};\lambda )\gamma_{j},$$

where *g*_*i*_ is the maximum channel conductance which scales the current amplitude (*S*/*F*); *γ*_*j*_ are gates of the channel determined by voltage-dependent forward and backward transition rates between open and closed states, *α* and *β*, characterized by gating parameters **λ**; *k*_*j*_ is an exponent term that may be applied to represent multiple identical gates in parallel; and *f* is some voltage-dependent forcing function (most commonly the difference between *V*_*m*_ and the ion Nernst potential). The gating equation may equivalently be transformed into a form explicitly specifying steady-state values, *γ*_∞_ and time constants, $\tau_{\gamma }$

2.2 $$\begin{array}{rl} \frac{\text{d}\gamma (t)}{\text{d}t}=\frac{\gamma_{\infty }(V_{m})-\gamma }{\tau_{\gamma }(V_{m})},\;\tau_{\gamma }(V_{m}) & ={\left[\alpha_{\gamma }(V_{m};\lambda )+\beta_{\gamma }(V_{m};\lambda )\right]}^{-1},\\ \gamma_{\infty }(V_{m}) & =\alpha_{\gamma }(V_{m};\lambda )\tau_{\gamma }\;\text{if activating gate},\\ \gamma_{\infty }(V_{m}) & =\beta \gamma (V_{m};\lambda )\tau_{\gamma }\;\text{if inactivating gate}, \end{array}$$

(where we omit the indexing subscript for clarity). There are no standard formulations for the voltage-dependent transition rates $\alpha_{\gamma }(V_{m};\lambda )$ and $\beta_{\gamma }(V_{m};\lambda )$, and each model implements a different set of equations [12,13]. For *I*_Na_, and for the C model also *I*_CaL_, the structure of these equations was inherited directly from the parent model (of a rabbit atrial cell [17] and guinea pig ventricular cell [18] for the N and C model, respectively), while *I*_to_, *I*_Kur_ and, for the N model, *I*_CaL_ were introduced as new formulations in each model. The equations are included in electronic supplementary material, S4. In this work, we are interested in the ability of the gating kinetics to reflect the experimental data, and the identifiability of parameters **λ** with respect to these data.

The standardized formulation, henceforth referred to as the S model, is used as a relatively simple baseline to compare to the more complex formulations in the N and C models. In this formulation, the transition rates between open and closed states have a structure based on free energy arguments [19,20], which have been shown as sufficient to capture the kinetics of a rapid delayed rectifier potassium current [16]. The transition rates are given by

2.3 $$\alpha (V_{m})=\lambda_{1}\exp(\lambda_{2}V_{m})\;\text{and}\;\beta (V_{m})=\lambda_{3}\exp(-\lambda_{4}V_{m}),$$

where the parameters requiring calibration are *λ*_1_, …, *λ*_4_ for each gate in the channel model. For *I*_Na_ and *I*_CaL_, which have two components of inactivation, we add another inactivation gate in parallel for the S model, which is related directly to the existing inactivation gate by a scale parameter on its magnitude, e.g. *τ*_*γ*2_ = *aτ*_*γ*1_, where *a* is the scale parameter. Only the activation gate of the S model for *I*_Na_ has a power of 3 applied to remain consistent with both N and C models.

### (b) Datasets and calibration

Parameters underlying gating kinetics are calibrated to experimental data from voltage patch-clamp experiments conducted on isolated cardiomyocytes. Though more complex protocols may be better able to explore the entire range of kinetics exhibited by different ion channels [16], the majority of available data were generated through the use of ‘traditional’ voltage stepping protocols. In these experiments, the transmembrane potential is held fixed and subsequently clamped to a series of voltage steps while the current across the membrane is recorded. Specific features of the recorded current can then be calculated and summarized across different cells or experiments.

Data from voltage patch-clamp experiments in human atrial myocytes for *I*_Na_ [21,22], *I*_CaL_ [23–25], *I*_to_ [26–28] and *I*_Kur_ [27,28] were digitized (including any error measurement). A virtual voltage-clamp protocol was created to replicate *in silico* each of the *in vitro* experiments. A full description of data sources and voltage-clamp protocols are included in electronic supplementary material, S1. The N model did not include calibration data for any time constants in *I*_Na_, for activation time constants in *I*_to_ and for deactivation of *I*_Kur_. The C model did not calibrate to voltage-dependent recovery data in *I*_CaL_ and *I*_Kur_. Neither model included an activation time constant measurement available for *I*_CaL_.

We use ABC to calibrate each channel model to the experimental data. ABC replaces an exact likelihood function by sampling parameter values from a chosen prior distribution and simulating the model under the specific voltage-clamp protocol. These simulated data are processed into summary statistics which can be compared to experimental data using a distance function. The prior distribution for each parameter is set to a uniform distribution. For the N and C models, the width of the prior is set based on the published value of the parameter and its position in the model structure. The width was increased if it was noted that during calibration the distribution was being restricted by the upper or lower prior limit. For the S model, the prior ranges were set as previously [16,29].

The summary statistics are calculated from the output of a function that makes specific measurements, for example peak current or decay rate from fitting an exponential equation, of the current trace in response to the voltage-clamp protocol replicated from the experimental publication. The summary statistic functions are assumed invariant to the magnitude of current, and thus channel conductance is not included as a calibration parameter. A low distance value generated by the distance function indicates that a particular sample from the parameter space is more likely to be from the ‘true’ distribution. This behaviour is captured algorithmically by using a threshold value which is used to decide whether to accept or reject a specific sample. Sunnåker *et al.* [7] provide a more detailed overview of ABC.

An advantage of ABC is prior knowledge of the experimental data can be embedded in the distance function during calibration. Data from voltage-clamp experiments include error bars to account for the different results from experimental repeats due to observational noise and other sources of experimental uncertainty [3]. We are more certain of the value of data points with low variance (small error bars) from the experimental datasets. To account for this, we use a weighted least-squares distance function with weights proportional to the inverse of the standard deviation at experimental data points. A regularization parameter is included to avoid divide-by-zero errors and set to 0.05. To avoid bias to individual experiments with more data points, this weighting is also proportional to the number of data points in an experiment. Further details are included in electronic supplementary material, S2.

### (c) Implementation

Voltage-clamp experiments were simulated using the myokit Python library [30]. The ABC sampling process uses the pyABC Python library [31] to implement the Toni ABC sampler based on sequential Monte Carlo [32]. In this sampler, the ABC process above is repeated through a number of iterations with reducing threshold value. Further details are included in electronic supplementary material, S2. We created the *ionchannelABC* Python library for applying ABC in this context which integrates pyABC and myokit for voltage patch-clamp ABC calibration (see Data accessibility).

When comparing the relative computational speed to solve different channel models, we apply a voltage pulse train protocol of 100 pulses (using channel-dependent voltage steps indicated in the text). We record the time taken for a simulation from each of the 100 samples from the posterior parameter distributions to account for variability.

To simulate the effect on the AP of re-calibrated channels, each new parametrization was inserted into either the entire N or C model. Channels were tested one at a time, and parameters in other channels left at their published values. One hundred samples were taken from the unified posterior distribution and a pulse train protocol applied to generate AP samples from the full model. The pulse train consisted of 1 ms current stimuli at a rate of 1 Hz and with amplitude 40 pA/pF. The AP elicited during the 100th pulse was recorded for analysis.

S channel models were then used in place of the published formulae to study the effect of a reduction in complexity on the overall AP. In all cases, the conductance of the channel was set by matching the peak current magnitude from each sample to the peak from the published channel model (peak current was assumed to occur at 60 mV for *I*_to_ and *I*_Kur_ models). This experiment could not be conducted for *I*_Na_ as both the N and C modellers positively shifted the steady-state curves according to macro measurements of the AP (such as velocity of the upstroke), and in the previous section, this channel was calibrated to the original experimental data. As in this study, voltage-clamp protocols were replicated exactly as described in the experimental publications; it is not clear how these would be adapted to fit the artificially shifted data, or how to manually shift the steady-state curves.

All figures display experimental measurements as mean ± standard deviation reported in the experimental publication. The calibrated model is displayed by taking 100 samples from the posterior distribution of parameters and plotting the output from simulations as median ± 89% high density posterior intervals (HDPI) [33].

## 3. Results

### (a) Existing gating parameter uncertainties

We first sought to study uncertainty and unidentifiability present in gating parameters of the existing models using the datasets originally cited for calibration [12,13]. Only *I*_Na_ and *I*_to_ channels of the C model are calibrated to the full range of data available (electronic supplementary material, table S1). It would be expected that a higher level of uncertainty is present in kinetics of the channel within voltage ranges that have not been explicitly tested. A high level of uncertainty in the parameter value is indicative of potential structural or practical unidentifiability.

**Figure 1.**
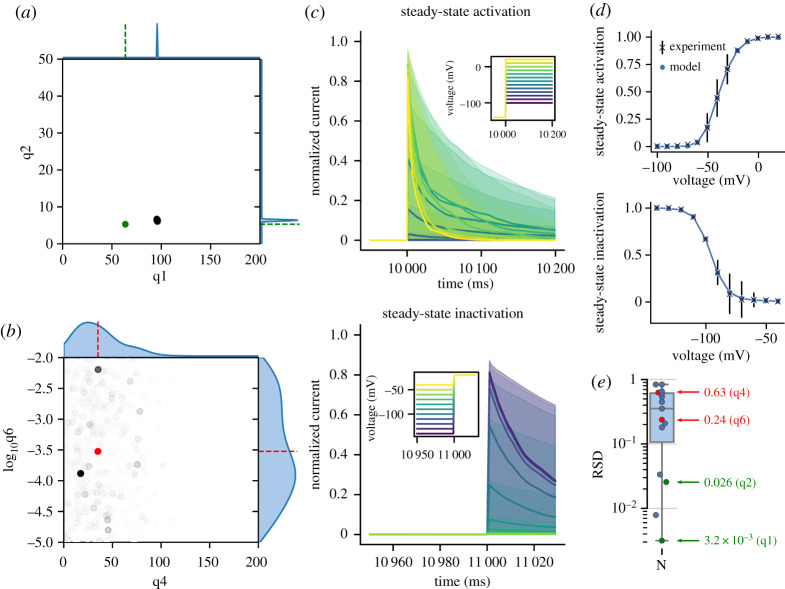
(*a*,*b*) Examples of parameter posterior distributions from the N model of *I*_Na_ after calibration to the original dataset which only included steady-state data. Scatter plots show the population of posterior ABC particles with weights indicated by opacity. One-dimensional KDEs of each parameter posterior distribution are displayed along the corresponding axes. The original parameter values from the model are indicated as dots and dashed lines. (*c*) Normalized current traces of posterior model response to inset voltage protocol displayed as median line with shading indicating 89% HDPI from 100 samples from parameter posterior distributions. The high variability in decay rate is the result of no time constant data included in the dataset used for calibration. (*d*) Summary statistics generated from results in (*c*). (*e*) RSD values of all parameter posteriors with representative parameters from (*a*) and (*b*) highlighted. (Online version in colour.)

For example, the *I*_Na_ channel in the N model was only directly compared to steady-state experimental data [21]. Figure 1*a*,*b* shows representative posterior distributions and kernel density estimates (KDEs) of those distributions following ABC calibration for this channel. Parameters underlying steady-state components of the channel (figure 1*a*) exhibited narrow posterior distributions indicating they were well constrained by the data. By contrast, parameters underlying time constants had relatively wide posterior distributions implying they are poorly constrained (figure 1*b*) and suggesting there is a higher level of uncertainty surrounding their value in the model (see electronic supplementary material, S4.1 for equations). The effect of the poorly constrained time constant parameters can be seen in figure 1*c*, which shows the response of the calibrated N model of *I*_Na_ to the voltage-clamp protocols. The decay rate of the current is highly variable, consistent with the observation of poorly constrained time constant parameters. Figure 1*d* shows how this uncertainty is ‘hidden’ by the steady-state summary statistics function used to process the traces in experiments. Note that the non-physiological error bars of the experimental data are a result of plotting as mean ± s.d.

Relative standard deviation (RSD; defined as *σ*/|μ|) is a scale-invariant measure of the width of the parameter posterior distributions and used to provide a comparison of the parameter uncertainty between models. Higher RSD values can indicate that a particular parameter is unidentifiable with respect to the model structure or available data. Figure 1*e* shows the RSD values for all parameter posterior distributions in the calibrated N model, and highlights the values for distributions shown in figure 1*a*,*b*. The parameters with narrower posterior distributions have RSD values orders of magnitude lower than those with wide posteriors (note the log scale on the *y*-axis). In this case, the four parameters with an RSD less than 10^−1^ can be interpreted as governing the shift and steepness of the steady-state activation and inactivation curves in figure 1*d*, and it is thus not surprising that these were more identifiable than parameters involved in rise and decay rates of the current.

### (b) Re-calibrating to a unified dataset

Having observed a range of poorly and well-constrained parameters when calibrating to the original datasets, we next sought to investigate the effect of re-calibrating each model to a different ‘unified’ dataset. This dataset is assembled from a union of the original experimental data sources.

**Figure 2.**
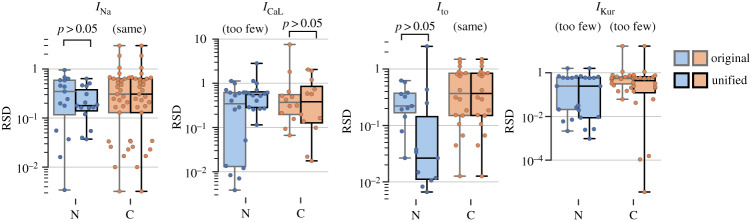
RSDs of each parameter in each channel model for original and unified experimental datasets in both N and C models. Significance tested with Wilcoxon signed-rank test. Text ‘too few’ above a pair of boxplots indicates that the sample size was too small to conduct the statistical test after eliminating differences of zero. (Online version in colour.)

Figure 2*a* shows the RSD of parameter posteriors for all channels studied in the N and C models when calibrated to the original and unified datasets. In all models, a wide range of RSD values is observed for the original datasets which confirms each model has a combination of parameters which are well defined and parameters which are potentially unidentifiable with respect to the given data. In the N model, no significant differences in the parameter posterior RSDs were observed between original and unified dataset calibrations for *I*_Na_ and *I*_to_ using a Wilcoxon signed-rank test. In both *I*_Na_ and *I*_CaL_, the minimum RSD increased after calibrating to the unified dataset. In *I*_CaL_ and *I*_to_, we also noted an increase in the maximum RSD. No significant differences were observed for the C model. Note that a Wilcoxon signed-rank test was not carried out for *I*_CaL_ (for the N model) or *I*_Kur_ as this statistical test requires discarding differences between pairs of zero and the resulting sample size was too small for a normal approximation. This is a result of only re-calibrating parameters of one gate to unified data in these models as the unified data for the other gate was the same as the original dataset (thus the RSD values of parameters in the other gate remains constant between the original and unified dataset).

Regions of high uncertainty in gating functions generally corresponded to behaviour or voltage ranges not tested by the experimental voltage-clamp protocols. Adding the additional data in the unified dataset generally reduced the variability in these regions, though often at the expense of other aspects of the model. For example, the N model of *I*_Na_, showed high uncertainty in the posterior estimates for time constant functions with the original dataset, which was reduced on calibration to the unified dataset. However, this reduction came at the expense of greater variability in the steady-state behaviour of the channel. A similar effect was noticed in the inactivation processes of the C model of *I*_CaL_. For the N model of *I*_to_ and the inactivation of the C model of *I*_Kur_, calibrating to the unified dataset resulted in noticeable changes to the shift and steepness of the steady-state functions. Full graphs of the posterior gating functions are included in electronic supplementary material, S3.1.

### (c) Comparing to a standardized model

No statistically significant reduction in RSD values for parameter posterior distributions was observed when re-calibrating the N and C models to more complete datasets. This implies that the additional data covering a wide range of kinetics for each channel was not sufficient to reduce unidentifiability observed in parameters. We hypothesized that this could be due to problems with structural unidentifiability caused by the complex form of the equations in either model. To test this, we next studied whether a simpler model structure of the S model could be used to reflect the same experimental data with reduced parameter uncertainty for each channel.

#### (i) Fast sodium channel

**Figure 3.**
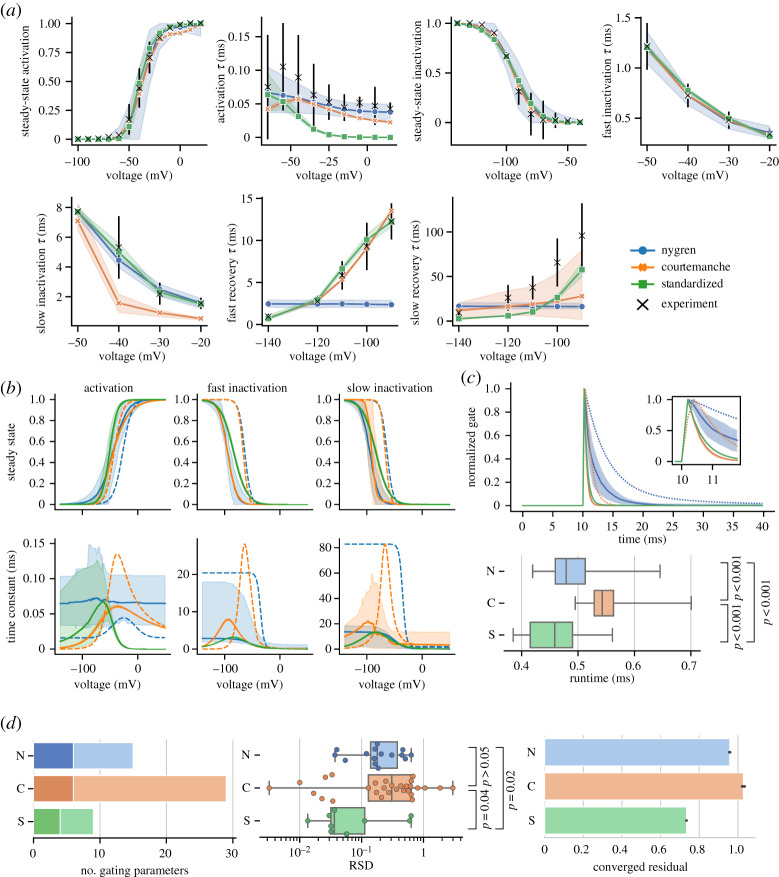
(*a*) Results of calibrating each *I*_Na_ model to the unified dataset. Model output is plotted as median line and 89% HDPI from 100 parameter posterior samples. Experimental data are plotted as black crosses and bars representing mean and s.d. (*b*) Steady-state and time constant functions for each gate from samples in (*a*). Dashed lines indicate the published N and C models. (*c*) Example traces from each model generated from the last step of a pulse train of 100 steps from −140 mV to −30 mV for 100 ms at a rate of 1 Hz using samples from (*a*). Dashed lines indicate the published N and C models. Boxplot compares time taken to run the protocol for each model. (*d*) Number of gating parameters in each model (left). Dark and light shading correspond to activation/inactivation gating parameters, respectively. RSD of parameter posteriors in each model (centre). Goodness of fit assessed by the converged residual of ABC (right). Significance tested using Mann–Whitney *U*-test. (Online version in colour.)

Figure 3*a* compares *I*_Na_ models calibrated to the unified experimental dataset. The goodness-of-fit of each model can be assessed by comparing the residual in the distance function after convergence, shown in the rightmost graph of figure 3*d* (a per-experiment version of this measure for all channels is given in electronic supplementary material, S3.5). In most experiments, the S model qualitatively reflects the experimental data to a comparable degree as the N and C models, improving notably over the N model in recovery experiments. The overall converged residual of the S model is 23.5% lower than the N model and 30.0% lower than the C model. It notably deviates from experimental data in the upper voltage range of the activation time constant where it falls too quickly towards zero. We observe that although the S model has in total nine parameters compared to 15 in the N model and 29 in the C model (figure 3*d*, left), the S model has more tightly constrained parameter posterior distributions exhibiting lower RSD (figure 3*d*, centre). This reduction of RSD values was statistically significant when tested using a Mann–Whitney *U*-test against the N model (*p* = 0.02) and C model (*p* = 0.04).

Figure 3*b* compares the underlying gate functions of each *I*_Na_ model. The S model exhibits generally well-constrained behaviour other than in the region of a gap in experimental data (deactivation data in lower voltages of the activation gate time constant). Only the C model exhibits low uncertainty in this region. In this case, the uncertainty may not be beneficial as it could imply undue confidence in the C model’s behaviour in model space without experimental data to compare. There is a distinct difference in the form of the N model inactivation gate time constants which have a sigmoid shape rather than the peaked curve exhibited by both C and S models. Comparing the current trace of each model at the end of a pulsetrain (figure 3*c*), there is little difference between the fully calibrated traces of the C and S models, and the S model has a significantly reduced runtime for this protocol.

#### (ii) L-type calcium channel

*I*_CaL_ has calcium- as well as voltage-dependent components of inactivation. The N and C models differ in how they formulate this channel; the former including two voltage-dependent inactivation gates and the latter including a single voltage-dependent inactivation gate. Both include a single calcium-dependent inactivation gate that was held at a constant value to isolate the voltage-dependent features of the channel. Given the data typically show a fast and slow component of inactivation [24], we include two voltage-dependent inactivation gates in the S model of *I*_CaL_. Thus comparisons are more meaningful between the N and S models in this case, as the structure of the C model differs substantially and relies more directly on intracellular calcium concentration to modulate the rate of current decay.

**Figure 4.**
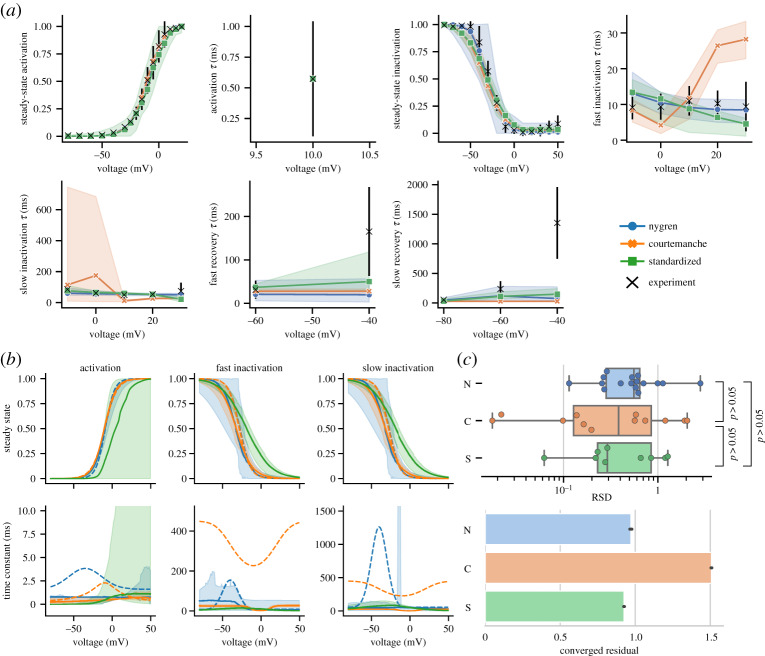
(*a*) Results of calibrating *I*_CaL_ models to the unified dataset. Plotted as described in figure 3. (*b*) Steady-state and time constant functions for each gate from the samples in (*a*). Dashed lines indicate published N and C models. (*c*) RSD of parameter posteriors (upper). Goodness of fit assessed by converged residuals from ABC (lower). (Online version in colour.)

Figure 4 summarizes the results for *I*_CaL_. None of the models appear able to calibrate to the slowest components of recovery from inactivation (figure 4*a*). The gating functions in figure 4*b* show high uncertainty in the steady-state function of the S model and differs from the N and C models at higher voltages of the inactivation steady-state curve. Each model has reduced uncertainty around the voltages of time constants which are explicitly tested (inactivation and recovery *τ* measurements) with, particularly in the N model, wide uncertainty outside of these ranges. Figure 4*c* shows there were no significant differences between RSD values between the models, highlighting that each model suffers from issues with parameter identifiability in some parts. The N and S models provided better fits to the data when assessed by the final converged residual though, as noted above, the C model relies more heavily on the calcium-dependent inactivation processes held constant in this experiment.

#### (iii) Potassium channels

**Figure 5.**
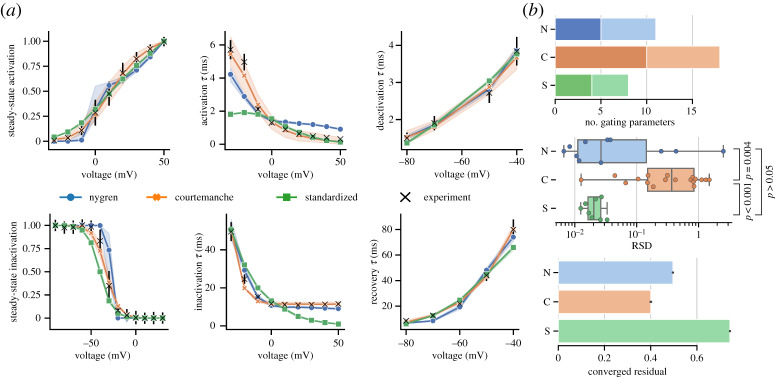
(*a*) Results of calibrating each *I*_to_ model to the unified dataset. Plotted as described in figure 3. (*b*) Number of gating parameters (top). RSD of parameter posteriors (centre). Goodness of fit assessed by converged residuals from ABC (bottom). (Online version in colour.)

Figure 5 summarizes the results from the calibration for *I*_to_. Although the N and S models both show parameter posteriors with significantly lower RSD values than the C model, this is balanced by the lower goodness-of-fit to the experimental data (figure 5*b*, lower). In particular, the S model was unable to capture kinetics such as the plateau region of the upper voltage range for the inactivation time constant. By contrast, the C model has the most parameters and appears to suffer from unidentifiability in a subset of these parameters (suggested by their high RSD values), but it also produces the best fit to the experimental dataset.

The underlying gating functions also reveal clear differences between models in the mid-point and slope of steady-state activation, and peak activation time constant (electronic supplementary material, figure S9). The peak time constants of each model are approximately at the mid-point voltage of activation and thus also differ from each other.

*I*_Kur_ exhibits a very slow voltage-dependent component of inactivation and only partially inactivates in the available voltage-clamp experimental data [28]. As a result of these factors, each channel model showed distinct differences when calibrated to experimental data using the experimental voltage protocols rather than comparing gating functions to experimental data directly. Each channel deviates from experiment data points at lower voltage ranges of steady-state activation, and the S model converged to a substantially different model output for the steady-state inactivation gate (electronic supplementary material, figure S10).

### (d) Effect on action potential

**Figure 6.**
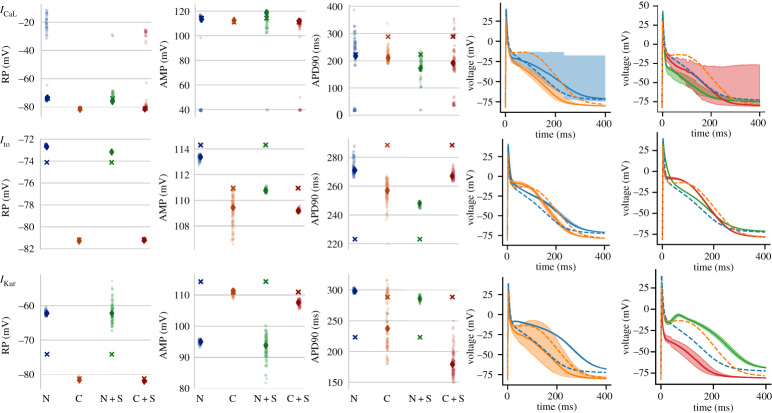
Strip plots showing measurements of resting potential (RP), action potential amplitude (AMP) and AP duration to 90% repolarization (APD90) from APs elicited from the full N and C models. Crosses indicate values from the models run at published settings. Each point is a measurement from an AP generated from a sample from the posterior parameter distribution of the channel indicated. The diamond is the median of the samples. N: using N unified posterior distribution in the full N model, C: using C unified posterior distribution in the full C model, +S: indicates the standardized model replaced the corresponding channel in the full model and used the S posterior distribution. Line plots in the two rightmost columns display a summary of the AP traces in each case. Traces are displayed as median lines with shading representing 89% HDPI from 100 posterior samples. The published N and C models are plotted as dashed lines in all plots for comparison. (Online version in colour.)

We next studied how the inclusion of uncertainty in the gating of these channels would impact the full AP of the cell models. Figure 6 shows how the AP changed for the channel models for the re-calibrated N channels, re-calibrated C channels, and inserting the re-calibrated S channel into either model (referred to as N+S and C+S). As noted in the Methods, this experiment could not be completed for *I*_Na_ models. More detailed results are in electronic supplementary material, S3.6.

For *I*_CaL_, a portion of posterior samples in the N model resulted in an elevated resting potential, though the median was still close to the published value. This causes the wide 89% HDPI observed in the corresponding trace, while the median line is much closer to the dashed line of the original model. This also occurred to a lesser degree when the S model inserted into the C cell model. The re-calibrated C model was more stable with the action potential duration (APD) being mainly reduced from the published value as a result of quicker and more complete inactivation of the channel during the AP (electronic supplementary material, figure 13B).

Traces generated by using the posterior parametrizations for *I*_to_ generally showed small differences from the published values for resting potential and AP amplitude, though larger changes in the APD. For the full N model (both with the original channel form and S form), an increased APD is observed while the opposite was observed for the C model. This makes the two AP models, which have quite different published AP morphology, more similar to one another. A small degree of variability is apparent from the posterior intervals around the traces and, in contrast to *I*_CaL_ all samples resulted in ‘normal’ APs.

Using the re-calibrated form of *I*_Kur_ resulted in more noticeable changes to the AP than *I*_to_. For both the N and N+S experiments, this increases the resting potential and AP amplitude accompanied by an increase in APD to a similar level as the published C model. In both cases, the triangular morphology of the published N model was altered to have a more prolonged plateau phase. For the C model, the resting potential and amplitude of the AP from close to the published values, while a large degree of variability is observed in the APD. When using the C form of the channel, this variability encompassed the published value, which is apparent from the shading of the AP trace including the dashed trace of the published C model. When using the S form of the channel, the cell repolarises more quickly as a result of an increased *I*_Kur_ current density.

## 4. Discussion

Understanding the uncertainty and unidentifiability of parameters in AP models is critical to the development of trustworthy cardiac models for the era of personalized medicine [3,5]. In this study, we have applied an ABC method based on sequential Monte Carlo to characterize the existing uncertainty in gating kinetics of four major ion channel types in human atrial cell models. The wide posterior distributions for a subset of parameters in all models was indicative of potential unidentifiability, which may be structural or practical. We then sought to assess whether the poorly constrained parameters could be more clearly identified by re-calibrating to complete datasets or through using a standardized gating formulation with less complex structure and fewer parameters to constrain. We finally looked at the changes introduced by using the re-calibrated *I*_CaL_, *I*_to_ and *I*_Kur_ channels in the full cell models.

Figure 1 shows uncertainty in parameter estimates for the *I*_Na_ channel current in the N model manifest in a range of possible outputs of the current trace in response to voltage steps. The medians of the posterior parameter distributions related to time constant behaviour were close to the original reported values but showed a high degree of uncertainty. This was the expected result as only steady-state data were used to calibrate *I*_Na_ of the N model. As a consequence, the time constant parameters in this example likely exhibit practical unidentifiability, as they cannot be constrained given the provided experimental data. It should be noted that the well-constrained parameter *q*_1_ in figure 1*a* is offset from the published value due to a constant offset applied to the steady-state curve in the N model justified by time- and/or temperature-dependent drifts in steady-state characteristics of the current [12].

In comparison, the C model used a more complete dataset to calibrate kinetics for this channel. Nevertheless, this model also exhibits parameters with high RSD values suggesting unidentifiability (figure 2). In contrast to the practical unidentifiability observed in the case of the N *I*_Na_ model, this is likely to be due to structural identifiability issues. The high number of parameters in the C *I*_Na_ model (figure 3*d*) facilitates over-fitting to experimental data and leads to redundancy of some parameters. In [4], Daly *et al*. investigated the uncertainty in parameter estimates of the Hodgkin–Huxley AP model. They reported wide posteriors around the estimates of certain parameters in the potassium and sodium channels of that far simpler AP model. It, therefore, is unsurprising to observe these results in more complex models of a sodium channel where there is more opportunity for parameters to covary within the structure of the model. Additionally, the use of conventional voltage-clamp protocols further aggravates this issue as it only provides an indirect measure of the underlying gating kinetics [34].

We next re-calibrated the N and C models to the unified datasets. In figure 2, it was apparent that the addition of data sources, most often relating to the time constant of gates, would reduce the uncertainty around the gating functions though often at the expense of increased uncertainty in other regions of the model behaviour. In the case of the N model of *I*_Na_, the addition of time constant data led to a reduction in the RSD value of time constant parameters at the expense of increased RSD values of the steady-state parameters. This may be a result of the structure of the N model *I*_Na_, with sigmoid functions for inactivation time constants, not providing a good fit to the experimental data. As a result, the ABC calibration reaches the sampling rate stopping criteria earlier because the steady-state experimental data now represent only a small portion of the overall calibration dataset.

The C model of *I*_Na_ has 29 parameters to constrain across three gates, the most of any of the channels investigated. Although using a complete dataset for calibration, the high RSD values of parameters show that this model still suffers from parameter unidentifiability. The complex form of the equations makes over-fitting possible when using conventional calibration processes such as simple least squares, which would not highlight the consequences of doing so as with a Bayesian method such as ABC. Often these complex forms of equations are initially based on direct comparisons between the gating functions and the experimental data and may be tailored to the specific data sources selected for calibration, rather than through forward evaluations of the model. In [34], the authors highlight the importance of replicating the experimental conditions and voltage protocols as closely as possible when calibrating AP models, despite the inherent difficulties of doing so. The complex form of model gating functions, which are often combined in parallel, means that the behaviour of the gating function itself may not be representative of the behaviour of the full channel model when tested with experimental protocols.

Reducing the structural complexity of the model equations by adopting a standardized gating formulation showed channel-dependent success. For *I*_Na_, the S formulation resulted in a significant reduction in RSD values for parameter posterior distributions (*p* = 0.02 compared to N model, *p* = 0.04 compared to C model), which suggests it is partly alleviating unidentifiability concerns. The *I*_Na_ S model output also gave a goodness-of-fit measure of converged residual lower than either other model (figure 3*d*). Despite this, for some experiments the goodness-of-fit was clearly worse than either N or C model, such as for activation and slow recovery time constants. In addition, there was variability around the lower voltage range of the activation gate time constant for which there is an absence of experimental data, highlighting this model is not immune to practical identifiability issues. For *I*_Na_, there appears to be an advantage to the less complex formulation which provides more confidence in the identifiability of its parameters without sacrificing representation of the experimental data. Fewer parameters also allow more direct reasoning about the effects of altering gating parameters on the overall channel behaviour. As with any model, considerations should to be taken of the specific goals of the modelling study, though the S *I*_Na_ presents a less complex foundation from which to build on with additional, context-specific data.

For *I*_CaL_, the S model produced approximately the same goodness-of-fit as the more complex N model. However, in this case, the similar RSD values across each model imply that the use of a simpler model structure did not alleviate issues relating to parameter unidentifiability. This is particularly noticeable in the steady-state activation gate of the channel, where there is a greater variation in the posterior behaviour compared to the other two models (figure 4*b*). This is potentially a result of the way steady-state summary statistics are calculated through normalizing to a reference value in the output. As a consequence, the gate is less constrained to be fully open at the maximum activation as the normalization hides this behaviour. The unidentifiability of parameters across all models for this channel is likely a consequence of the relative paucity of experimental data relating to the voltage-dependent time constant behaviour of *I*_CaL_, which has fewer voltages tested in the unified dataset compared to the other channels studied.

For both potassium channel models (*I*_to_ and *I*_Kur_), simplifying the model structure is a balance between reduced unidentifiability of parameters and reduced goodness-of-fit to experimental data. The inactivation *τ* plot of figure 5*a* shows an example where both the N and C models are able to capture the plateau region at upper voltage ranges while the S model tends to zero. Similarly, the form of the S model requires the peak of the time constant curve to occur at the mid-point of activation or inactivation which appears inappropriate for the activation gate of this channel. This is reflected in the higher converged residual of the S model compared to the N and C models (figure 5*b*, lower). The parameters of the less complex model have lower RSD values than the C model (*p* < 0.001) and a smaller range than the N model. This suggests that the closest fit of this model to the experimental data is identifiable, despite also being a poorer fit than either other model. This highlights the fact that low uncertainty in posterior parameters does not necessary imply a model fits the data well (and vice versa), and a trade-off exists which may depend on the goals and particular use-case of a modelling study.

Beattie *et al.* [16] used the same standardized gating formulation to model the behaviour of a rapid delayed rectifier potassium current using an information-rich voltage protocol. In their case, the studied current appears to satisfy the requirement of peak time constant of the gate at mid-point of the steady-state curve (see, for example, fig. 5 in [16]). Based on these observations and our results, this standardized approach may, therefore, be appropriate in cases when experimental data suggests particular requirements, such as this one, are met. Using a standard gating formulation alleviates problems associated with the high number of parameters in very detailed models, without sacrificing the biophysical basis for the model. In contrast to purely phenomenological models, the form of the S model is based on Eyring-derived transition rates giving its parameters a physical interpretation [16,19].

The S model leads to a reduction in simulation times, which is an important consideration as patient-specific modelling is further explored in whole-heart tissue simulations. However, in contrast to simpler models which have been shown to reproduce patient-specific AP morphologies [35], the standardized formulation retains information about specific ion channel currents which provides a stronger body of evidence in terms of model validation. It would be encouraging if future ion channel modelling promoted the use of common forms of equations in cell models rather than the current heritage of complex equations. Another promising proposition is a model reductionist approach to reduce uncertainty in parameter estimates by eliminating parameters which have little effect on the model output, e.g. manifold boundary approximation [14].

When testing the new parametrizations in the full AP models, it was apparent that a proportion of samples led to non-physiological behaviour. This was also the case when combining samples from all new parametrizations, which generally caused simulations to fail. This highlights the importance of a feedback process in the development of AP models, where the form of the full AP also informs the design of the underlying currents. A future step for this work could follow a similar approach to Kernik *et al.* [36] with multiple stages of calibration. For example, the full AP samples in these results could be used to further constrain the posterior distribution of the channel model parameters by eliminating non-physiological cases. These results also demonstrate an important consideration in the development of full AP models that tends to be omitted from modelling papers: the sheer challenge and achievement of combining a variety of nonlinear models of individual channels usually developed in isolation into a single model of a cardiac cell and tuning to produce a physiological AP.

### (a) Limitations

RSD was used as a measure of the width of parameter posterior distributions and as an indicator of parameter unidentifiability in this study. This measure tends to become inflated when the mean value in the denominator is close to zero. This approach does not allow us to separate structural and practical unidentifiability and the likely form was inferred from the availability of experimental data and structural form of the model in experiments.

Our ABC stopping criterion for all experiments was set to halt execution once an iteration had dropped below a 1% particle acceptance rate. This was based on preliminary experiments on the C model where it was assumed the algorithm is close to the optimum solution once sampling became too difficult. However, in some cases, this criterion may be excessive or insufficient, as was observed for example for the original N and S model of the *I*_CaL_ channel. Investigations into more appropriate stopping criterion were outside the scope of this work. Parameters involved in the calcium-dependent gate in *I*_CaL_ of all models were omitted from calibration and the gate set to a constant value due to the lack of specific data and difficulty in isolating calcium handling components of the cell model. Particularly for the C model, which relies on the calcium transient to modulate the inactivation rates, it is perhaps inappropriate to attempt calibration using this approach.

The calibration process relied on summary statistics of the model responses to the virtual voltage-clamp protocols. Reducing the data in this fashion was necessary to obtain the same form as in the experimental dataset. However, as highlighted in [7], it is generally not possible to obtain a finite-dimensional set of summary statistics that are sufficient to fully capture all relevant information obtained from a voltage-clamp protocol.

Channel models with greater than 14 parameters (N: *I*_CaL_, *I*_Kur_; C: *I*_Na_, *I*_to_, *I*_Kur_) could not be calibrated as a complete model due to the large number of particles required to sample the high-dimensional parameter hyperspace. In these cases, we calibrated the behaviour of parameter subsets for each gate separately to the relevant experimental data while leaving the remainder at their published values. It is possible that the original gates could affect the calibration of the chosen gate, for example in exponential fitting to decay traces for channels with fast and slow inactivation. Despite this, it should be noted that conventional calibration techniques (e.g. least-squares regression) do not restrict the modeller from applying the method in the case of this kind of sparse sampling space and will not explicitly convey the implications of doing so.

### (b) Conclusion

In this work, we have applied ABC to re-calibrate the gating kinetics in detailed ion channel models of human atrial myocytes. We calibrated these models to the experimental datasets used in the published calibration and showed a portion of parameters exhibited wide posterior distributions indicative of unidentifiability. Calibration to more complete experimental datasets did not reduce the unidentifiability present, which suggested that it may be both structural and practical. Reducing the structural complexity of the model through a common gating form was successful in reducing unidentifiability in *I*_Na_ without sacrificing goodness-of-fit. Experiments with other channels suggested that a trade-off exists between tailoring a model to provide a good fit to experimental data, and identifiability of parameters as models become more complex. The technique employed in this work is general and could be applied to any model of an AP.

## Supplementary Material

Supplementary Material

## Data accessibility

Electronic supplementary material is available with the article. All code is available in the project repository at https://github.com/charleshouston/ion-channel-ABC under the human-atrial project. Results database files: Dryad https://dx.doi.org/10.5061/dryad.p2ngf1vmc [37].
