# Supplementary material for: Reducing complexity and unidentifiability when modelling human atrial cells
Source: Philos Trans A Math Phys Eng Sci. 2020 May 25;378(2173):20190339. doi: 10.1098/rsta.2019.0339 (PMC7287336; doi:10.1098/rsta.2019.0339)
Supplement: Supplementary Material [file rsta20190339supp1.pdf]

Supplementary material for Houston et al., 2020, Reducing complexity and unidentifiability when modelling human atrial cells, *Phil. Trans. R. Soc. A.* doi: 10.1098/rsta.2019.0339

Contents

S1 Datasets and simulations **2**
S1.1 Data sources . . . 2
S1.2 Temperature adjustment . . . 3
S1.3 Voltage-clamp protocols and summary statistic functions . . . 5
S1.3.1 I_{Na} . . . 5
S1.3.2 I_{CaL} . . . 6
S1.3.3 I_{to} . . . 8
S1.3.4 I_{Kur} . . . 10

S2 Approximate Bayesian computation **12**

S3 Additional results **13**
S3.1 Gating functions for calibrations to original and unified datasets . . . 13
S3.2 I_{CaL} . . . 15
S3.3 I_{to} . . . 15
S3.4 I_{Kur} . . . 16
S3.5 Goodness-of-fit residuals . . . 17
S3.6 Action potential response . . . 18

S4 Model equations and numerical results **25**
S4.1 I_{Na} . . . 25
S4.2 I_{CaL} . . . 29
S4.3 I_{to} . . . 34
S4.4 I_{Kur} . . . 38

©The Authors under the terms of the Creative Commons Attribution License http://creativecommons.org/licenses/by/3.0/, which permits unrestricted use, provided the original author and source are credited.

S1 Datasets and simulations

S1.1 Data sources

Table 1 contains a complete list of all experimental data sources for both original and unified datasets.

Channel	Gate		Data	N [1]	C [2]	Unified
I_{Na}	activation, m	m_∞	Fig. 2 [3]	✓	✓	✓
		τ_m	Fig. 3C [4]	✗	✓	✓
	inactivation*, $\{h,j\}$	$\{h,j\}_\infty$	Fig. 7 [3]	✓	✓	✓
		$\tau_{\{h,j\}}$ (inact.)	Fig. 5B [3]	✗	✓	✓
		$\tau_{\{h,j\}}$ (recov.)	Fig. 9 [3]	✗	✓	✓
I_{CaL}	activation, d	d_∞	Fig. 5C [5][a]<br>Fig. 2B [6][b]	✓[a]	✓[b]	✓[b]
		τ_d	Pg. H233 [6]	✗	✗	✓
	inactivation†, f	f_∞	Fig. 2B [6]	✓	✓	✓
		τ_f (inact.)	Fig. 3B [6][a]<br>Fig. 4B [7][b]	✓[a]	✓[b]	✓[a]
		τ_f (recov.)	Pg. H230 [6]	✓	✗	✓
I_{to}	activation, r	r_∞	Fig. 3A [8][a]<br>Fig. 2A [9][b]	✓[a]	✓[b]	✓[b]
		τ_r (act.)	Fig. 5D [2]‡	✗	✓	✓
		τ_r (deact.)	Fig. 5D [2]‡	✗	✓	✓
	inactivation, s	s_∞	Fig. 3C [10][a]<br>Fig. 2C [9][b]	✓[a]	✓[b]	✓[b]
		τ_s (inact.)	Fig. 4C [1][a,‡]<br>Fig. 5D [2][b,‡]	✓[a]	✓[b]	✓[b]
		τ_s (recov.)	Fig. 4C [1][a,‡]<br>Fig. 5D [2][b,‡]	✓[a]	✓[b]	✓[b]
I_{Kur}*	activation, a	a_∞	Fig. 8E [9]	✓	✓	✓
		τ_a (act.)	Fig. 8F [9]	✓	✓	✓
		τ_a (deact.)	Fig. 5B [2]‡	✗	✓	✓
	inactivation, i	i_∞	Fig. 3C [10][a,§]<br>Fig. 7A [9][b,§]	✓[a]	✓[b]	✓[a]
		τ_i (inact.)	Fig. 4D [1][a,‡]<br>Fig. 5B [2][b,‡]	✓[a]	✓[b]	✓[a]
		τ_i (recov.)	Fig. 4D [1]‡	✓	✗	✓

Supplementary Table 1: Summary of patch clamp experimental datasets used in modelling papers in human atrial myocytes for each channel studied. Ticks and crosses are used to indicate which datasets are included in the original model calibration and which compose the unified dataset. *There are some differences in the terminology used in each model. The N model refers to the inactivation gates of I_{Na} as h_1 and h_2, and the I_{Kur} channel as I_{sus}. †The L-type calcium current has a calcium-dependent inactivation process which is not calibrated in this experiment (discussed further in results). ‡In some cases it was not clear from the modelling paper where the comparison data plotted were obtained from. In these cases, the data points from the modelling paper itself are used and a protocol assumed based on the experimental paper cited. §In some cases it was not explicit which figure among a number of possibilities within a cited data source was used; the choice was inferred from the modelling paper.

S1.2 Temperature adjustment

The N model was created to simulate a human atrial action potential at 306.15K ($33°C$), whereas the C model was created to simulate at 310K ($\sim 37°C$). Time constant measurements of rise and decay rates are temperature-dependent, and thus it was important to account for this during the calibration. During ABC, time constant measurements from the experimental sources were adjusted to the temperature of the model being calibrated using a Q10 factor from an experimental source. The Q10 factors used are: I_{Na}: 2.79 [11]; I_{CaL}: 1.7 (activation), 1.3 (inactivation), both calculated from values in [6]; $I_{\{\mathrm{to,Kur}\}}$: 2.2 [9]. In the original publication for the C model, I_{to} and I_{Kur} were fitted at room temperature (295.15K) and then adjusted by the authors to the model temperature (310K) by dividing time constants by a factor of 3 [2]. To maintain consistency, we also calibrate the C model at room temperature rather than make any adjustment to the experimental data. The time constants for this channel are adjusted before being used to simulate a full action potential. The S model was always calibrated by adjusting experimental data to 310K. Figure 1 shows the temperature-adjusted datasets for all experiments across channel models.

When comparing channel models on the same figure throughout this work, their time constants are adjusted for the same temperature of 310K. For full action potential simulations, the N and C model time constants are kept at their model temperature, and only the S model time constant adjusted from 310K to 306.15K when it is added to the N model.

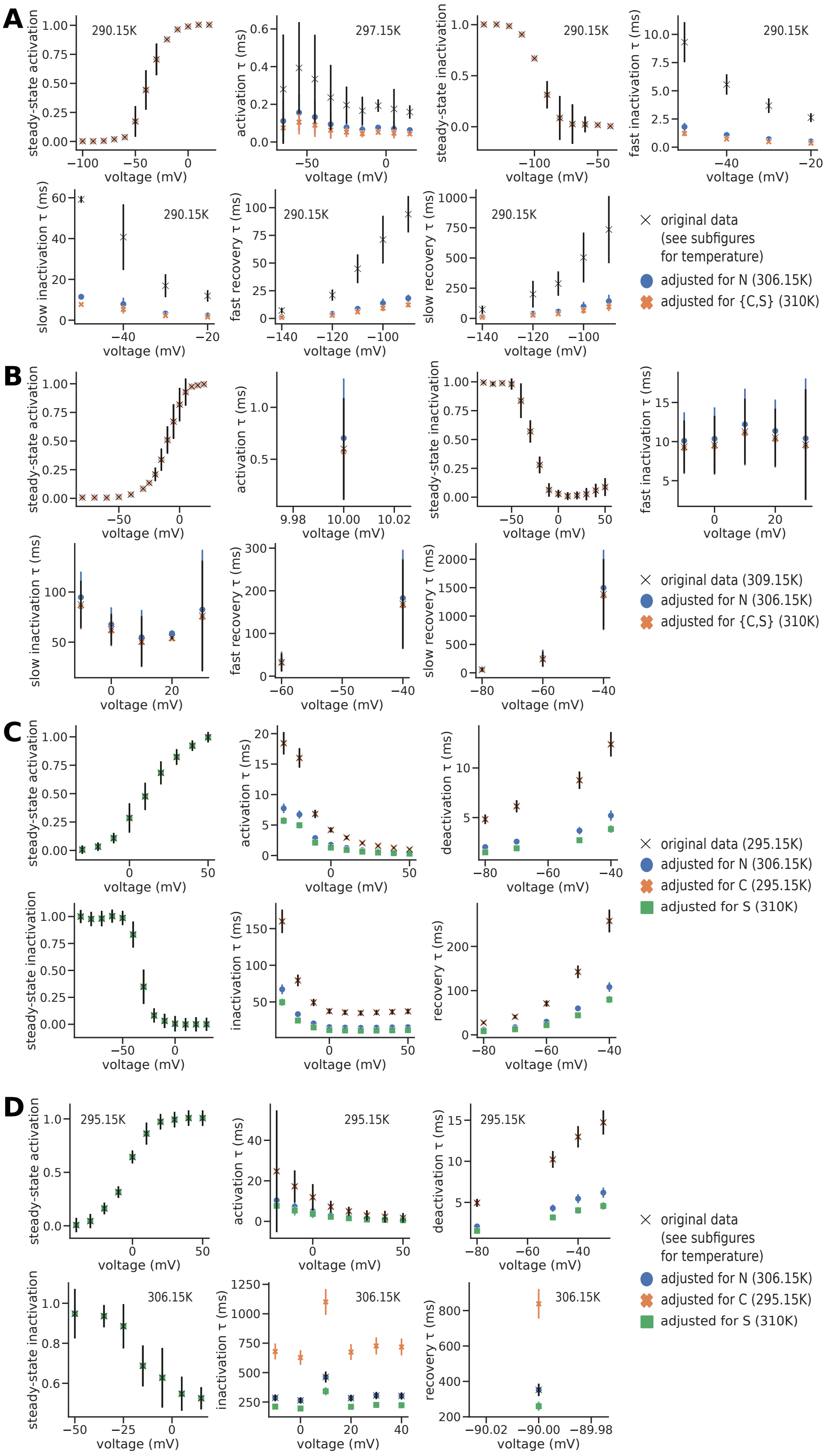

Supplementary Figure 1: Unified datasets for each channel model showing adjustments to experimental calibrating data at each model temperature. The adjustment is made using a Q10 factor as indicated in the text. **A** I_{Na}. **B** I_{CaL}. **C** I_{to}. **D** I_{Kur}.

S1.3 Voltage-clamp protocols and summary statistic functions

Throughout this section, the lettering in the headers refers to the figure describing voltage protocols for the channel. All curve fitting for finding time constants was carried out using the *scipy.optimize* Python library.

S1.3.1 I_{Na}

All experiments by Sakakibara et al. [3] used equal extracellular and pipette solution sodium concentration of 5mM at a temperature of 290K (17°C). The time constant of activation experiment from Schneider et al. [4] used sodium concentration of 120mM in the extracellular solution and 70mM in the pipette solution, and the experiment was conducted at 297K (24°C).

A: Steady-state activation (p.538 [3]). The standard protocol to measure steady-state activation of the channel holds the membrane at a sub-threshold potential of −140mV and then steps the channel to a series of voltage steps between −100mV and 20mV, with intervals of 10mV. The steps last for 1s and there are 10s between each step.

The degree of steady-state activation is measured by recording the peak current during the voltage step (normalised to cell capacitance). The conductance is calculated by dividing the peak current by the forcing term, usually assumed to be the potential difference to the Nernst potential of the primary ion carrier, $g = \frac{I}{V - E_{\mathrm{X}}}$. In the computational protocols, we directly measure the conductance of the channel to bypass this calculation. To plot the activation curve, the conductance is plotted against the voltage step normalised to its maximum value in any voltage step.

B: Time constant of activation (p.85 [4]). The time constant constant of inactivation is measured by fitting an equation to the current trace from a standard steady-state activation protocol as described in the previous protocol. In this case, the holding potential is −135mV and the steps are from −65mV and 15mV in steps of 10mV. The time at holding potential between each step was not given and so assumed to be 10s (more than enough for the I_{Na} channel to return to steady-state) and each test pulse lasted for 12ms.

The activation time constant was measured by fitting the entire current trace at a pulse to the equation $I_{\mathrm{Na}} = I_{\mathrm{Na,max}} \left[1 - e^{-t/\tau_{\mathrm{m}}}\right]^3 e^{-t/\tau_{\mathrm{h}}} + \text{constant}$ (p.87 [4]). In this equation, t is the time in ms, $I_{\mathrm{Na,max}}$ is the peak of the current trace, τ_{m} is the activation time constant and τ_{h} is the inactivation time constant.

C: Steady-state inactivation (p.541 [3]). The protocol used to measure steady-state inactivation is often also referred to as an availability protocol. The membrane is held at a holding potential of −140mV for 10s, then stepped to a conditioning potential for 1s to activate and inactivate the channel. The membrane potential is returned to the holding potential for 2ms before stepping to a test pulse at −20mV for 30ms. A series of different conditioning pulses between −140mV and −40mV in steps of 10mV is used to test the amount of inactivation of the channel at different voltages.

The steady-state inactivation is measured by recording the peak current during the test pulse, normalised to the current in a test pulse when no conditioning step is applied (usually the maximum current amplitude). In the virtual voltage clamp experiment, we measure conductance directly in this step (which is equivalent as the forcing is the same during each test pulse and eliminated during the normalisation).

D: Fast/slow inactivation time constant (p.539 [3]). The protocol to determine inactivation time constants is a simple steptrain of test pulses from a holding potential of −140mV for 10s to a series of 100ms test pulses to voltages from −50mV to −20mV in steps of 10mV.

The fast and slow inactivation constants are determined by fitting the *decay* part of the current trace (after the peak current) to the equation $I_{\mathrm{Na}} = A_1 e^{-t/\tau_{\mathrm{f}}} + A_2 e^{-t/\tau_{\mathrm{s}}} + A_0$ (p.538 [3]) where A are amplitude variables, t is the time and τ_{f} and τ_{s} are the fast and slow time constants of inactivation respectively.

E: Fast/slow recovery time constant (p.538 [3]). The time constants of recovery from inactivation were determined using a double pulse protocol. The first pulse is a conditioning pulse for 1000ms to −20mV followed by a recovery period to a holding potential between −140mV and −90mV in steps of 10mV, of varying length between $2 - 1000$ms (this was not specified and we assumed a series of $t_{\mathrm{r}} = 2^i$ where $i = 1, 2...10$). The recovery period is followed by a test pulse identical to the conditioning pulse. We assumed 10s at holding potential between each pair of pulses.

The recovery time constant is measured through a series of processing steps. Firstly, the peak current in the test pulse is normalised to the peak current in the preceding conditioning pulse to give a measurement of proportion of the channel recovery for each recovery time period. This recovery measure is plotted against the recovery time period and the resulting curve is fit to a double exponential equation $r = A_0 - A_1 e^{-t_{\mathrm{r}}/\tau_{\mathrm{r(f)}}} - A_2 e^{-t_{\mathrm{r}}/\tau_{\mathrm{r(s)}}}$ where r is the proportion of recovery, A are amplitude parameters, t

is the recovery time period and $\tau_{\mathrm{r(f)}}$ and $\tau_{\mathrm{r(s)}}$ are the fast and slow recovery time constants respectively. These values are calculated for each holding potential.

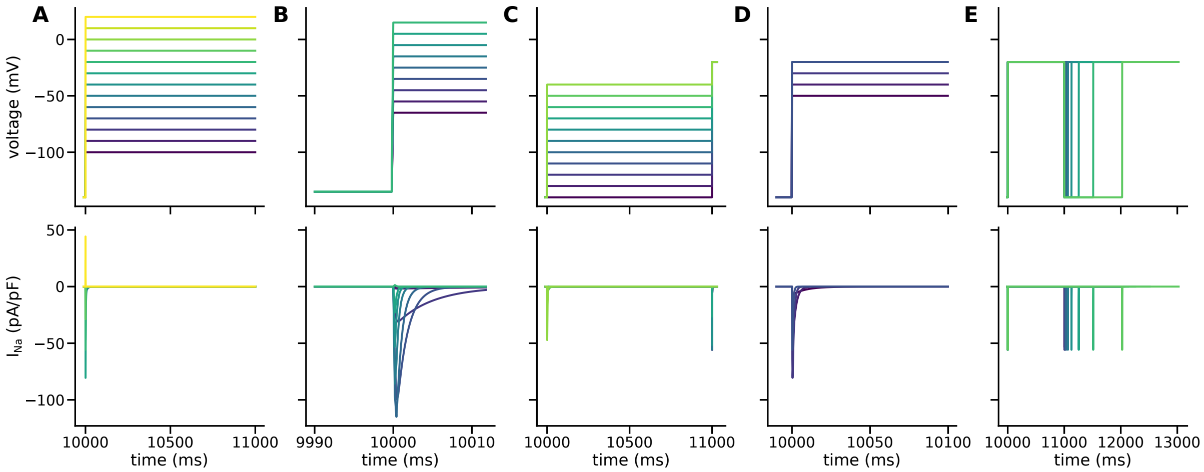

Supplementary Figure 2: Voltage steps and current response (from C model) of I_{Na} protocols. A: steady-state activation, B: time constant of activation, C: steady-state inactivation, D: fast and slow time constants of inactivation, E: fast and slow time constants of recovery from inactivation. The recovery protocol is repeated at multiple holding potentials (only one shown). See text for details of how the current traces are processed into summary statistics.

S1.3.2 I_{CaL}

Experiments by Mewes and Ravens [5] were carried out at room temperature (assumed by authors to be 295K) and external solution with calcium concentration of 1.8mM. Those by Li and Nattel [6] were conducted at 309K with 2.0mM external calcium concentration. Experiments by Sun et al. [7] were completed at room temperature (assumed by authors to be 296K) and external calcium concentration of 1mM. It is difficult to estimate the level of intracellular calcium concentration during these experiments due to the mechanisms of the intracellular calcium stores and buffering. In our virtual voltage clamp experiments, the level of the intracellular calcium was kept constant at the resting value from the published N and C models (72.5nM and 101.3nM respectively) and, for the S model, set to the same value as the C model (101.3nM).

A, B: Steady-state activation (p.1309 [5], p.H228 [6]). In Mewes and Ravens [5], steady-state activation was assessed using a conventional steptrain protocol from a holding potential of −40mV to 450ms steps between −35mV to 15mV in intervals of 5mV, with 10s between each pulse. In Li and Nattel [6], the activation curve was generated from the IV curve dataset which used a similar step train protocol. This time the holding potential was −80mV and the 300ms test pulses ranged from −80mV to 20mV in steps of 10mV. Both activation curves were generated as described in I_{Na} steady-state activation.

B: Activation time constant (p.H233 [6]). The single activation time constant value was determined from the current trace evoked during the 10mV test pulse in the activation protocol from [6]. The activation time constant was estimated by fitting the upstroke of the normalised current trace to $I_{\mathrm{CaL}} = 1 - Ae^{-t/\tau_{\mathrm{a}}}$ where A is an amplitude parameter, t is the time and τ_{a} is the activation time constant.

C: Steady-state voltage-dependent inactivation (p.H229 [6]). The voltage-dependent steady-state inactivation was assessed using a standard availability protocol. Conditioning pulses to a series of voltages between −80mV and 50mV in steps of 10mV were followed immediately by a test pulse to 10mV for 300ms. In [6], there are three datasets using different length of conditioning pulses of either 150ms, 300ms or 1000ms. We use the 1000ms prepulse dataset as this was used for calibration in both modelling papers. The inactivation curve was calculated as in I_{Na} steady-state inactivation.

D, E: Fast/slow voltage-dependent inactivation time constant (p.H229-H230 [6], p.H1628 [7]). Fast and slow inactivation time constants were calculated by fitting a biexponential equation to the decay portion of the current trace during test pulses from a holding potential. In [6], three different holding potentials were used and we use the same as the modelling papers: −80mV. Test pulses lasted 300ms

to steps between -10mV and 30mV in intervals of 10mV (we assume 10s between pulses). In [7], the holding potential was -80mV, test pulses had duration 1000ms with the same levels as above, and were preceded by a 500ms pulse to -40mV. In both cases, time constants of inactivation were calculated by fitting the decay portion of the current trace during the test pulses to $I_{\mathrm{CaL}} = A_0 + A_f e^{-t/\tau_{\mathrm{f}}} + A_s e^{-t/\tau_{\mathrm{s}}}$ were A are amplitude parameters, t is the time and τ_{f} and τ_{s} are the fast and slow time constants respectively.

F: Fast/slow voltage-dependent recovery time constant (p.H229-H230 [6]). Recovery time constants were assessed with a two-pulse protocol as in I_{Na} recovery experiments. In this case, the holding potentials were -80mV, -60mV and -40mV with conditioning and test pulses both to 10mV for 300ms. Recovery periods were generated from $t_{\mathrm{r}} = 2^i$ where $i = 1, 2, ..., 11$ based on the range of data points in Fig 4B [6]. The data was processed as in I_{Na} recovery experiments with the exception that a single exponential function was used to fit the -80mV recovery curve to give a single (slow) recovery time constant.

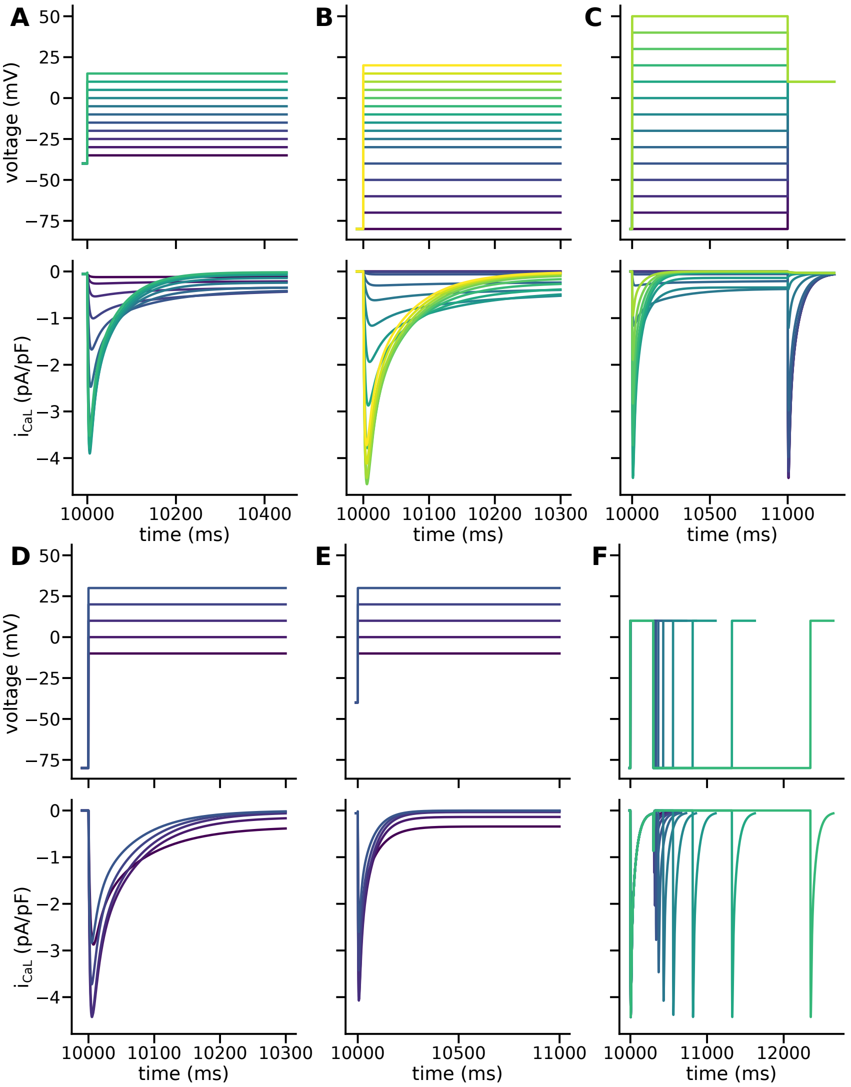

Supplementary Figure 3: Voltage steps and current response (from N model) of I_{CaL} protocols. A: steady-state activation [5], B: steady-state and time constant of activation [6], C:steady-state inactivation [6], D: fast and slow time constants of inactivation [6], E: fast and slow time constants of inactivation [7], F: fast and slow time constants of recovery from inactivation [6]. The recovery protocol is repeated at multiple holding potentials (only one shown). See text for details of how the current traces are processed into summary statistics.

S1.3.3 I_{to}

Experiments by Shibata et al. [8] and Wang et al. [9] were conducted at room temperature (assumed to be 295K). Firek and Giles [10] used temperature of 306K ($33°C$). Shibata et al. used an extracellular and pipette potassium concentration of 4.5mM and 150mM respectively, Wang et al. used 5.4mM and 130mM and Firek et al. used 5.4mM and 140mM.

Some data for I_{to} was extracted directly from the N and C modelling papers [1, 2] as the source of the comparison experimental data plotted was not clear from the text. In these cases, conditions were assumed to be the same as the lab which produced the model. Thus, conditions for the assumed experimental data from Nygren et al. [1] was set to the same conditions as Firek and Giles [10] above, and data from Courtemanche et al. [2] was assumed to have been collected at the conditions in Wang et al. [9].

A, B: Steady-state activation (p. H1776 [8], p.1065 [9]). In Shibata et al. [8], steady-state activation was determined by holding at a potential of −60mV for 20s, then depolarising for 15ms to a step between −30mV to 80mV, and finally stepping to −40mV for 100ms. The activation curve was generated by recording the peak current amplitude in the final step (the 'tail' current). Wang et al. [9] used a standard steady-state activation protocol with a holding potential of −80mV to a series of test potentials between −40mV and 50mV and measured the peak current during the 1000ms depolarising step (before processing as above for I_{Na}).

C: Activation time constant (p.H305 [2]). Activation time constant data corresponding to that plotted in the modelling paper could not be found in the cited experimental source [9]. A simple steptrain protocol as described in [9] was assumed with a holding potential of −50mV and 100ms test pulses. The activaton time course of I_{to} was fitted to a single exponential equation: $I_{to} = A_0 - Ae^{-t/\tau_a}$ where A parameters are amplitudes, t is the time course and τ_a is the activation time constant.

D: Deactivation time constant (p.H305 [2]). The deactivation time constant data source is also uncertain and it is assumed to use a similar protocol as in Fig. 9A in Wang et al. [9]. This protocol holds the membrane potential at −50mV for 20s before applying a 10ms conditioning pulse to 50mV. This is followed by a test pulse to elicit a tail current, which is fit to a single exponential (same as the activation time constant above) to determine the deactivation time constant.

E, F: Steady-state inactivation (p.34 [10], p.1065 [9]). In Firek and Giles, a standard steady-state availability protocol was applied as described in more detail in I_{Na} steady-state inactivation. The holding potential is −80mV, followed by a 400ms conditioning pulse to levels between −80mV and 16mV, and finally a 400ms test pulse to 0mV to activate the outward current. Wang et al. [9] similarly uses a standard availability protocol with the same holding potential followed by 1000ms conditioning pulse to a range of voltages between −90mV and 30mV followed by a 1000ms test pulse to 60mV. Output was processed into summary statistics as described in I_{Na} steady-state inactivation.

G, H: Inactivation time constant (p.66 [1], p.H305 [2]). It was not clear in either N or C model where experimental comparison data of time constants of inactivation for I_{to} were obtained from. Consequently, simple protocols were assumed based on single-pulse protocols in experimental papers originating from the same labs. For [1], a single pulse protocol from a holding potential of −80mV to 400ms test pulses between 0mV and 40mV was applied and the decay phase of the current trace fit to a single exponential equation as above for activation time constants. For [2], a similar protocol was assumed based on [9] with a holding potential of −50mV and 100ms test pulses to between −40mV and 50mV.

I, J: Recovery time constant (p.66 [1], p.H305 [2]). Similarly to the inactivation time constant data, it was unclear where the recovery data was obtained from for both N and C models. For the N model, we assume the recovery protocol in [8] was used. This is a standard two-pulse recovery protocol with holding potentials of −100mV, −80mV and −60mv and 100ms test pulses to 20mV. For the C model, we assume the protocol in [9] was used with holding potential between −60mV and 40mV and 200ms test pulses to 50mV.

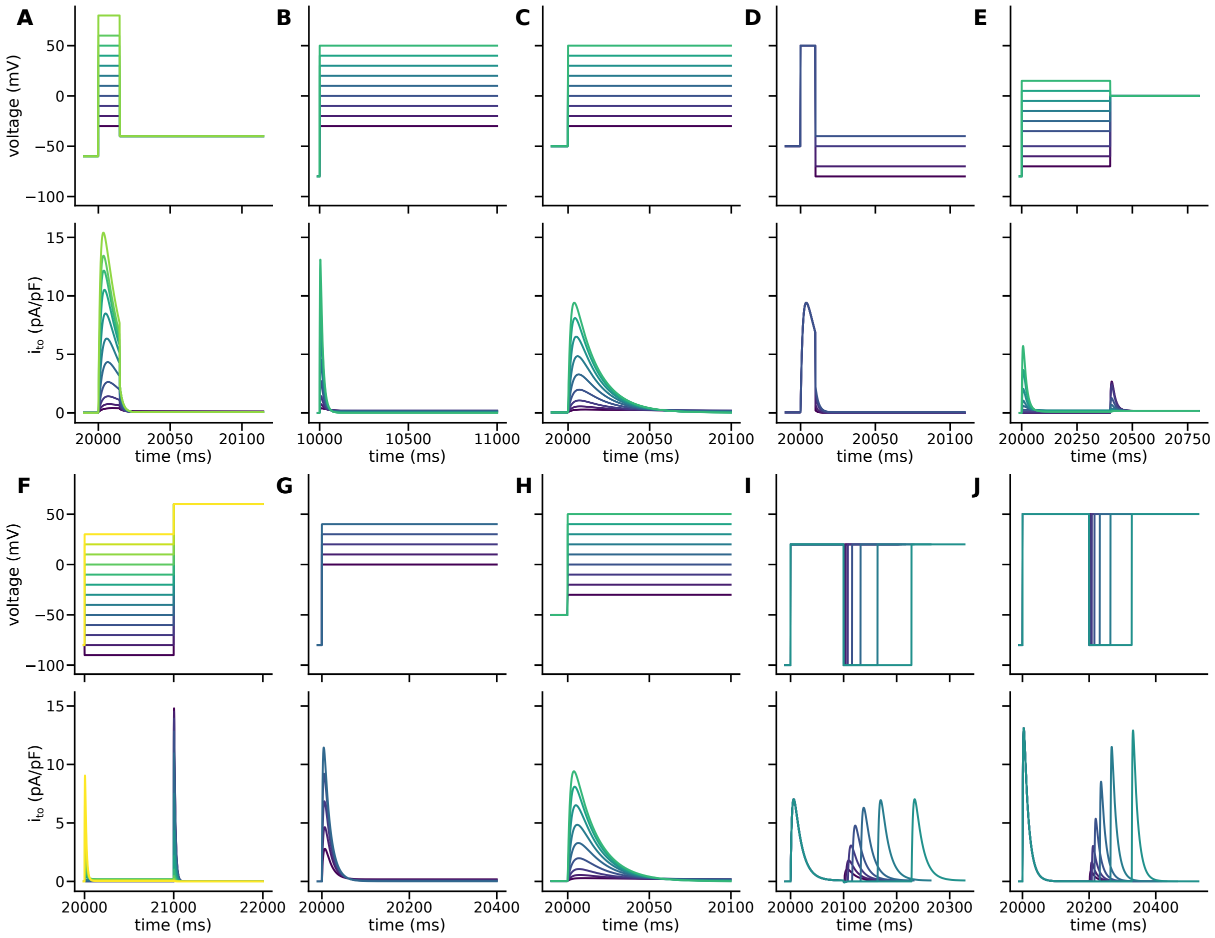

Supplementary Figure 4: Voltage steps and current response (from N model) of all I_{to} protocols. From left to right: A: steady-state activation [8], B: steady-state activation [9], C: activation time constants [2], D: deactivation time constants [2], E: steady-state inactivation [10], F: steady-state inactivation [9], G: time constant of inactivation [1], H: time constant of inactivation [2], I: time constant of recovery from inactivation [1], J: time constant of recovery from inactivation [2]. The recovery protocols are repeated at multiple holding potentials (only one shown for each). See text for details of how the current traces are processed into summary statistics.

S1.3.4 I_{Kur}

Experiments by Wang et al. [9] and Firek and Giles [10] use the same conditions stated above for I_{to}. As before, in some cases it was unclear where the comparison data in the modelling papers was obtained from. Details are given in the following sections.

A: Steady-state activation, activation time constant (p.1069 [9]). I_{Kur} was measured from a holding potential of −50mV followed by a 1s prepulse to 50mV (experimentally used to inactivate the I_{to} current which would otherwise interfere with isolating I_{Kur}). The potential is returned to −50mV for 20ms before being stepped to a range of 100ms test pulses between −40mV to 50mV each followed by a repolarising pulse to −10mV. I_{Kur} was measured as the peak current in the final repolarising pulse and the activation curve determined as previously described for I_{Na} steady-state activation. The activation time constants were determined by fitting the time course of I_{Kur} trace during the test pulses to a single exponential function as described above.

B: Deactivation time constant (p.305 [2]). As it was not clear where the data points were obtained from, the protocol from Fig. 9 in [9] was assumed to have been used. This is the same protocol as described in deactivation time constant for I_{to}.

C, D: Steady-state inactivation (p.34 [10], p.1068 [9]). For Firek and Giles [10], the protocol was the same as the steady-state inactivation protocol described for steady-state inactivation of I_{to} with an increase of the length of the conditioning pulse from 400ms to 2500ms. I_{Kur} was measured as the steady-state current at the end of the test pulse. In Wang et al. [9], steady-state inactivation is measured from the steady-state current at the end of a 2000ms test pulse to 40mV after a 1000ms conditioning pulse from a range of voltages. The holding potential was −60mV.

E, F: Inactivation time constant (p.66 [1], p.H305 [2]). It was unclear where the experimental data points from the N model paper came from. We assume they are generated from the protocol used in [10] from the same lab. This protocol is a simple 400ms step to a range of potentials from a holding potential of −80mV. In [10], the current decay is fit to a double exponential equation in order to separate I_{to} and I_{Kur} decay rates. As in the virtual voltage clamp there is only I_{Kur}, we fit the current decay to a single exponential.

Similary for the C model, we assume a similar protocol as in [9] was used. This is a simple 2000ms step to a range of test potentials from a holding potential of −50mV. The decay portion of the current trace is fit to a single exponential function as above.

G: Recovery time constant (p.66 [1]). As previously for I_{to}, we assume that the recovery protocol from [8] was used to determine a recovery time constant for I_{Kur}. The protocol is as described for I_{to} recovery time constant.

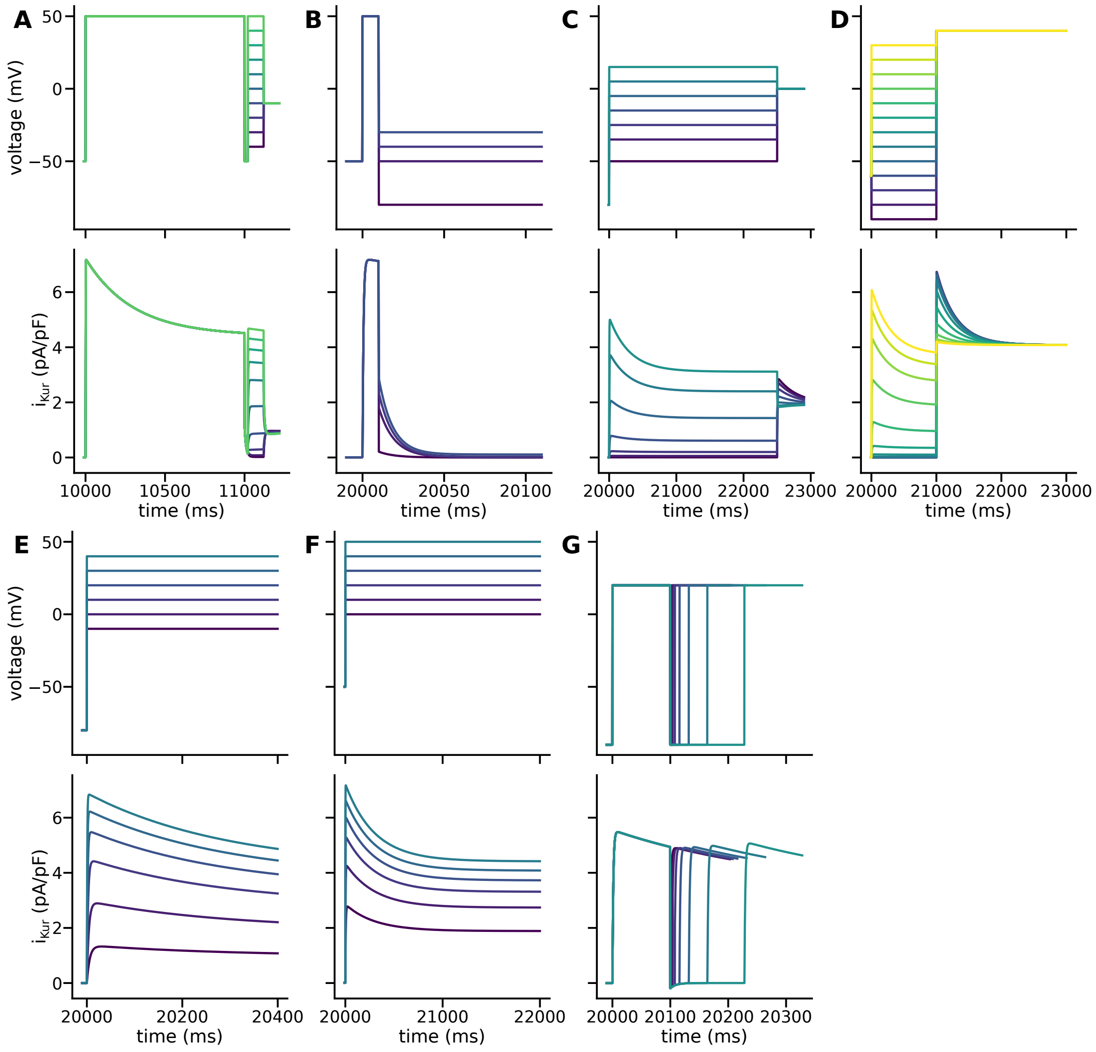

Supplementary Figure 5: Voltage steps and current response (from N model) of all I_{Kur} protocols. A: steady-state activation and activation time constant [9], B: deactivation time constants [2], C: steady-state inactivation [10], D: steady-state inactivation [9], E: time constant of inactivation [1], F: time constant of inactivation [2], G: time constant of recovery from inactivation [1]. See text for details of how the current traces are processed into summary statistics.

S2 Approximate Bayesian computation

Formally, ABC approximates the true parameter posterior distribution $P(\boldsymbol{\lambda}|\boldsymbol{D})$ by $P(\boldsymbol{\lambda}|\rho(\hat{\boldsymbol{D}},\boldsymbol{D}) \leq \epsilon)$ where $\boldsymbol{D}$ are the experimental data, ρ is the chosen distance function, $\hat{\boldsymbol{D}}$ is the model summary statistics and ϵ is the threshold value. We use the Toni ABC sampler based on sequential Monte Carlo to infer our parameter posterior distributions [12]. In this sampler, the ABC process above is repeated through a number of iterations with reducing ϵ. A population of parameter samples, referred to as 'particles', are propagated through each iteration and represent a discrete surrogate to the continuous posterior distribution.

At each iteration of the algorithm, the previous population of particles are perturbed slightly, by a multivariate Gaussian kernel, and used as the prior distribution for the current iteration. ϵ is reduced over iterations and chosen as the median distance of samples from the previous iteration. The particle population number is set according to the number of parameters being constrained in the experiment by considering the size of the parameter sampling hyperspace and assuming at least two particles in each dimension. A limit of 10000 particles is enforced due to computational demands. The algorithm terminates when less than 1% of parameter samples are accepted in a given iteration indicating the algorithm is struggling to improve on the current optimum. This criterion is chosen over termination at an absolute value of the distance metric termination used in other studies due to differences in number of model parameters and availability of data between experiments.

To compare our model summary statistics to the experimental data (which include error measurements at each point), we use a weighted Euclidean distance function

$$\begin{gathered} \rho(\hat{\boldsymbol{D}},\boldsymbol{D}) = \left[\sum_{i=0}^{M}\left(\frac{\hat{D}_i - D_i}{w_i}\right)^2\right]^{1/2}, \\ w_i = \max\left(\sigma_i, \delta\right) n_{\exp|i}, \end{gathered} \tag{1}$$

where w_i are the weights for each data point, σ_i is the standard deviation associated with the error at each data point, δ is a regularisation factor applied when weights are close to zero, and $n_{\exp|i}$ is the number of data points within the experiment. In this work δ was set to 0.05. Once generated, the weights are also mean-normalised to improve convergence of the ABC algorithm. The choice of distance function reduces the weighting of data points based on the magnitude of experimental uncertainty. This allows us to use information of which experimental data points we are relatively more confident about during the model calibration, propagating some of the experimental uncertainties through to this stage in the model development [13]. In some cases for I_{to} and I_{Kur}, it was not clear in the modelling paper where the data were obtained. In these cases, the points displayed in the figure from the modelling paper were digitised and 10% standard deviation error assumed (based on the error of similar measurements given in [9]).

Additionally, we account for the fact that we are simultaneously calibrating to multiple datasets by weighting according to the number of data points in a specific experiment. This provides balance between the different types of channel behaviour, rather than favouring an experiment with a greater number of data points. Each dataset is normalised to the maximum value in that experiment to avoid preference towards datasets with measurements at a larger scale in the ABC loss function.

A uniform prior distribution is used for each model parameter in the first iteration. The width of this prior is set to be sufficiently wide to cover the range of physiological possibilities. After convergence, the parameter posterior distributions are inspected and, if observed to be restricted by the lower or upper limit of the prior, the calibration is re-started with a wider prior. For the S model, prior ranges are set as in [14].

S3 Additional results

S3.1 Gating functions for calibrations to original and unified datasets

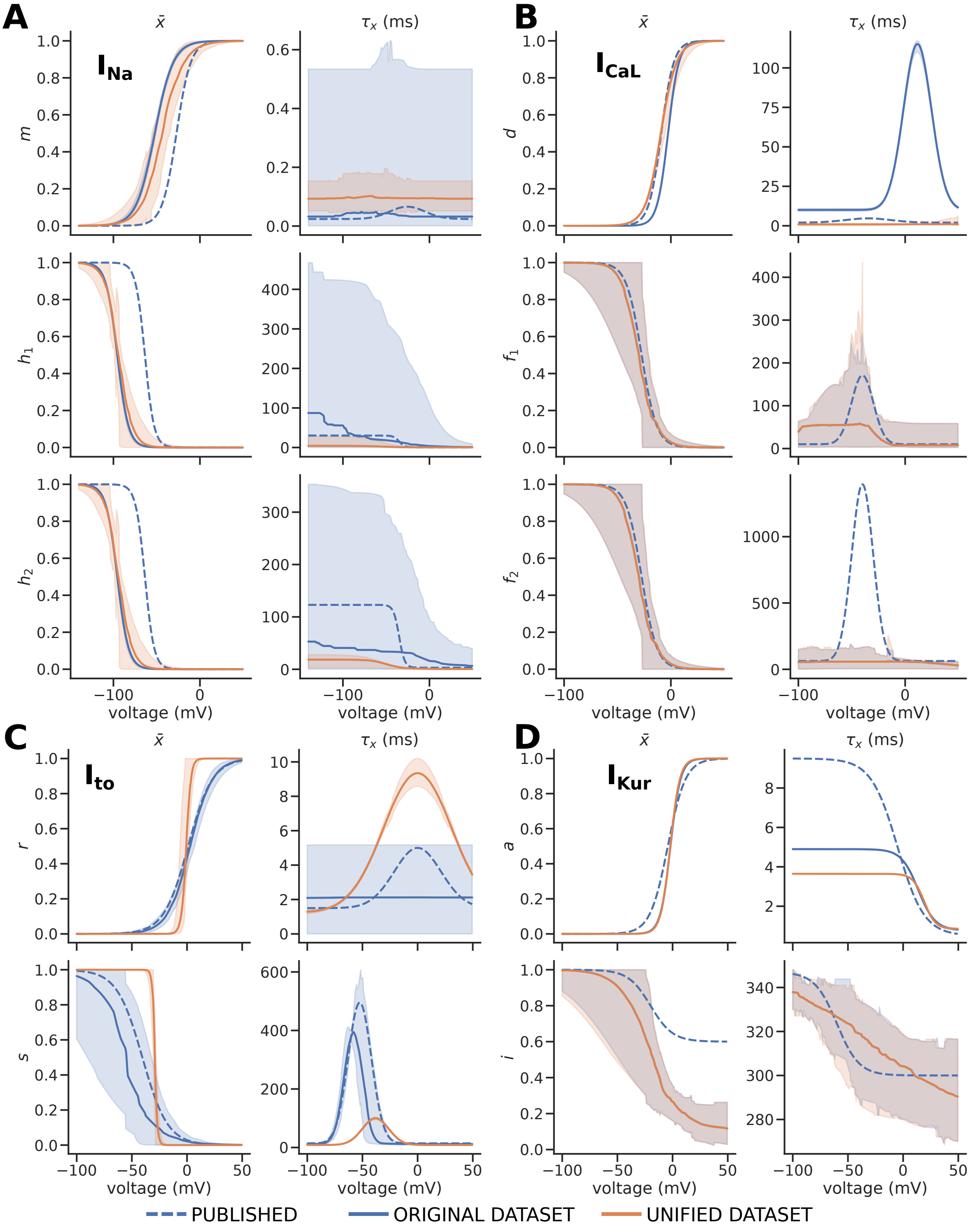

Supplementary Figure 6: A-D Steady-state and time constant functions for each channel of the N model using original calibration dataset and unified dataset. Blue refers to original dataset and orange to unified dataset. Data displayed as median line with shading representing 89% HDPI of 100 samples from the parameter posterior distribution.

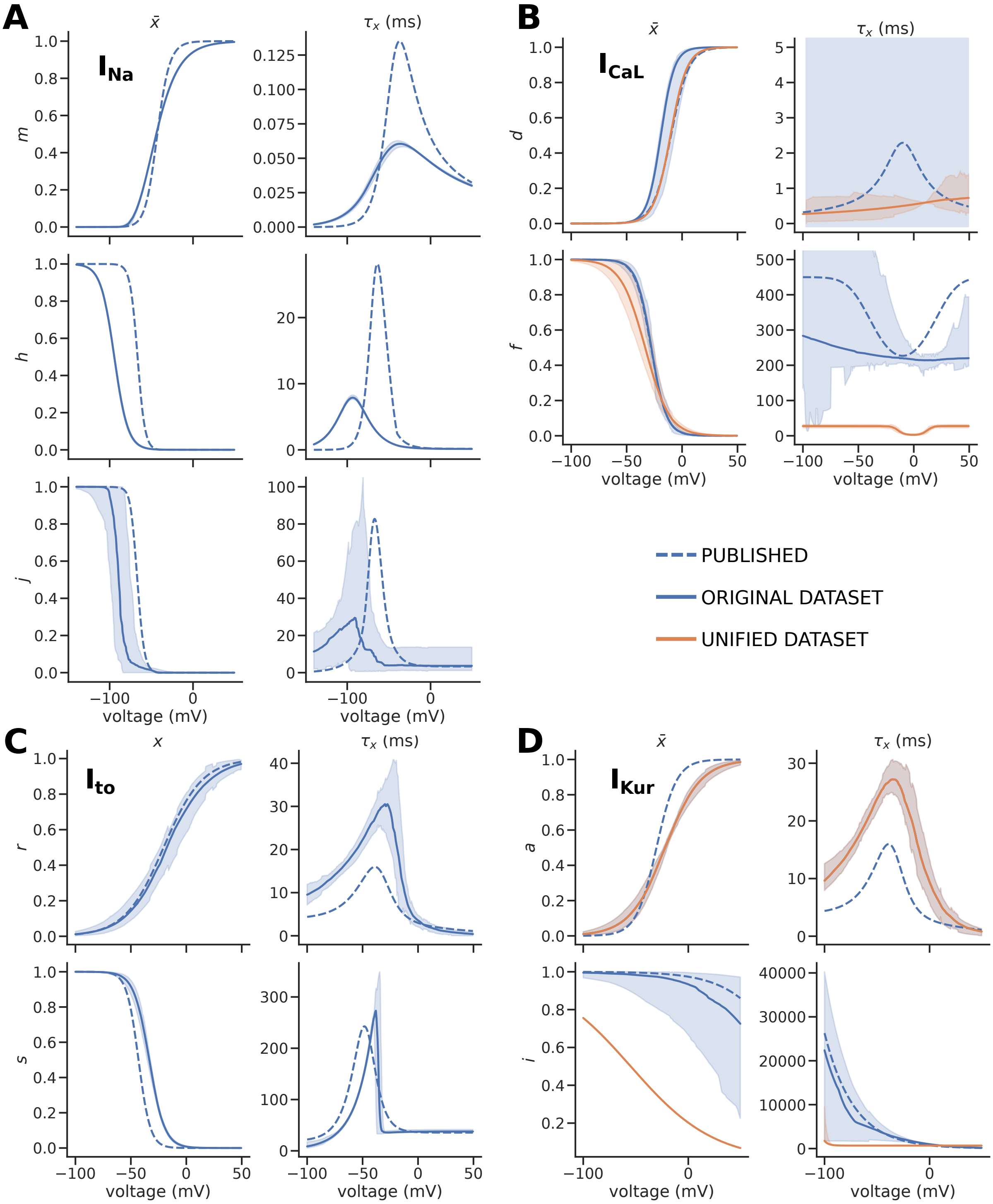

Supplementary Figure 7: A-D Steady-state and time constant functions for each channel of the C model using original calibration dataset and unified dataset. Blue refers to original dataset and orange to unified dataset. Data displayed as median line with shading representing 89% HDPI of 100 samples from the parameter posterior distribution. Note for I_{Na} and I_{to} the original and unified dataset are the same.

S3.2 I_{CaL}

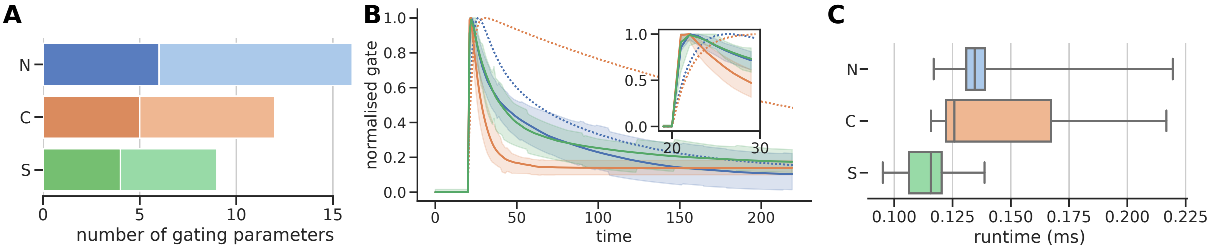

Supplementary Figure 8: **A** Number of gating parameters in equations for each I_{CaL} model, separated into activation (dark) and inactivation (light). **B** Example traces from each model generated from the last step of a train of 100 steps from -80 mV to -10 mV for 200 ms at a rate of 1 Hz using 100 samples from the parameter posterior distributions. Higher detail of the activation portion of the trace is shown in the inset plot. Output is summarised as median line with shading representing 89% HDPI. Dashed lines indicate the response of the published N and C models. **C** Boxplot comparing runtime of the simulation to generate each trace in B for each model.

S3.3 I_{to}

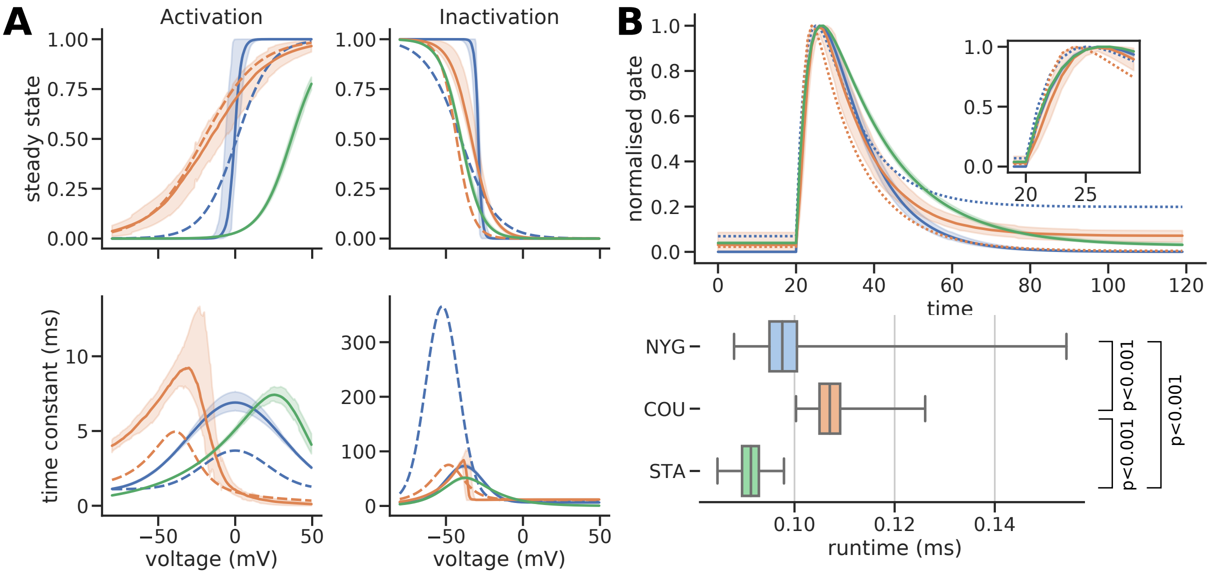

Supplementary Figure 9: **A** Steady-state and time constant functions for I_{to} model. Plotted as median line with shading indicated 89% HDPI from 100 samples from the parameter posterior distribution. Dashed lines indicate the published values. **B** Example traces from each model generated from the last step of a pulse train of 100 steps from -50 mV to -10 mV for 100 ms at a rate of 1 Hz using samples in A. Higher detail of activation portion of trace is shown in inset. Dashed lines indicate the published N and C models. The boxplot below compares runtime of the simulations to generate the above traces for each model.

S3.4 I_{Kur}

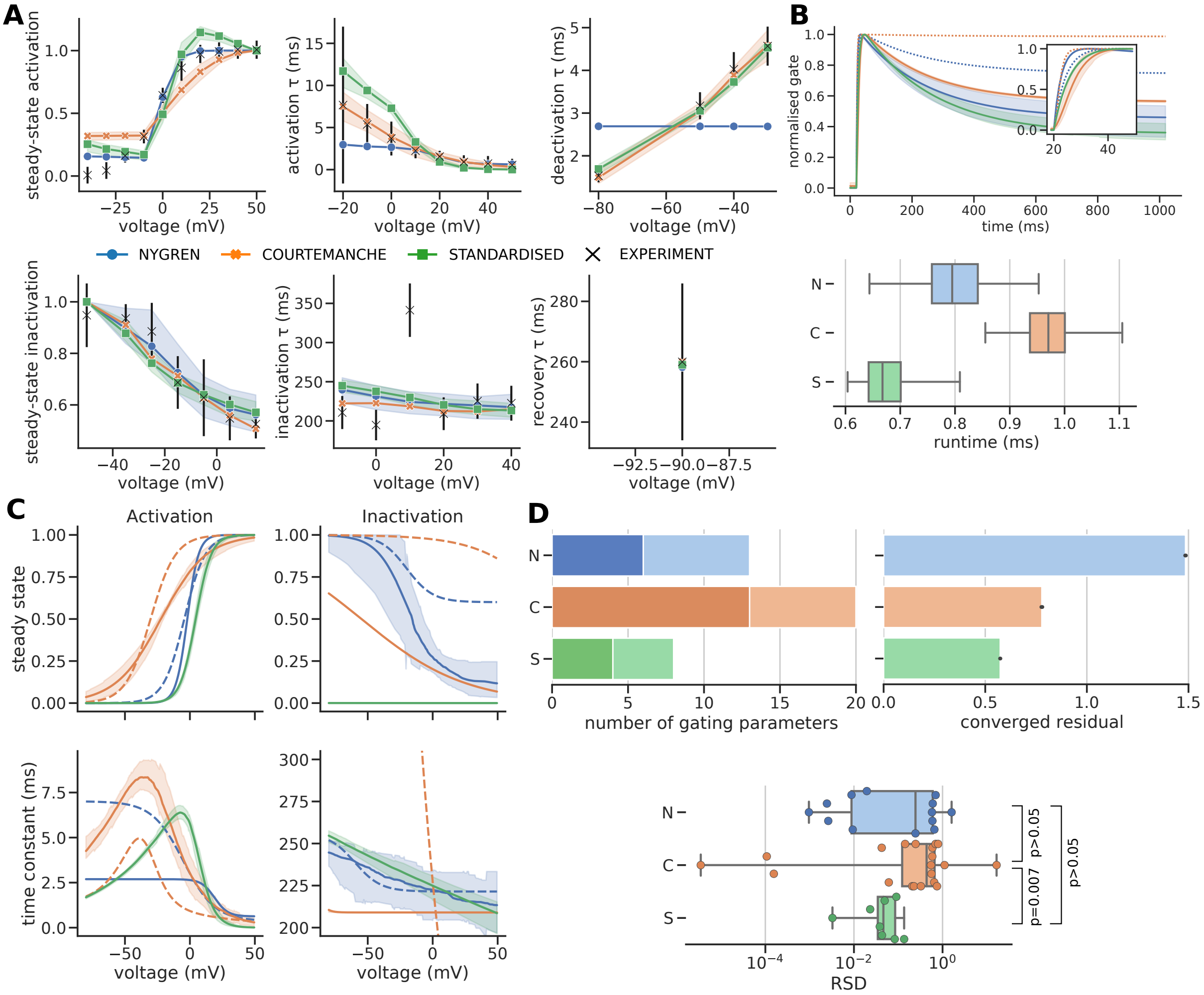

Supplementary Figure 10: **A** Results of calibrating each I_{Kur} model to the unified dataset. Model output is plotted as median with shading representing 89% HDPI generated from 100 samples from parameter posterior distributions. **B** Example traces from each model generated from the last step of a pulse train of 100 steps from -50 mV to -10 mV for 1000 ms at a rate of 0.1 Hz using samples from A. Dashed lines indicate the original N and C models. Higher detail of activation portion of trace is shown in inset plot. Boxplot compares runtime of simulations to generate the traces above for each model. **C** Steady-state and time constant functions for each gate generated from samples in A displayed as median lines with shading showing 89% HDPI. Dashed lines indicate original N and C models. **D** Number of gating parameters (top-left). Dark and light shading correspond to activation gate and inactivation gate parameters respectively. Goodness of fit assessed by converged residuals from ABC (top-right). RSD of parameter posteriors (bottom). Mann-Whitney U-test used to test significance.

S3.5 Goodness-of-fit residuals

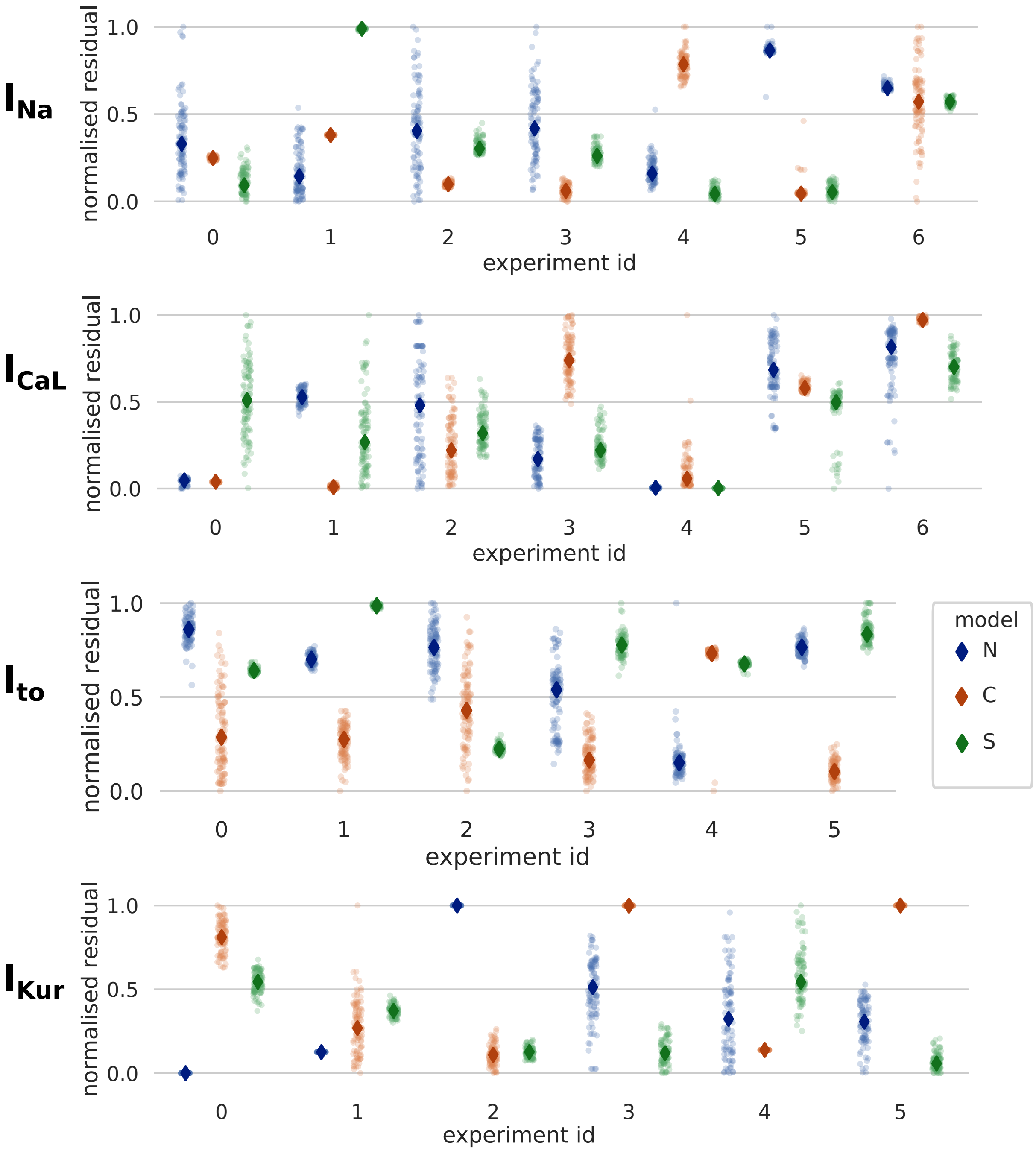

Supplementary Figure 11: Min-max normalised residuals by experiment and model. Experiment ID follows order of appearance in main figure for that channel.

S3.6 Action potential response

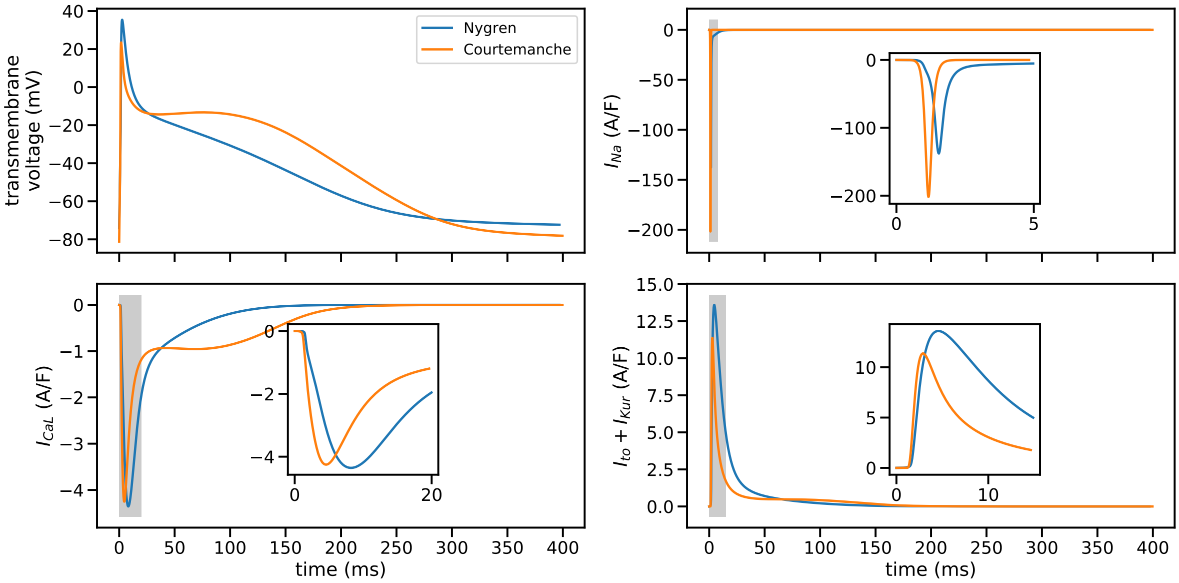

Supplementary Figure 12: Comparison of action potential morphology and major ion currents underlying the action potential in published N and C models of the human atrial cardiomyocyte. Inset graphs show more detail of the shaded time portion in the main graphs. Action potentials were stimulated by 100s of pacing at a basic cycle length of 1 s using a stimulus current of 40 pA/pF for 1 ms. The plot shows the final pulse from the pulse train protocol.

Model	Measure (units)	Published	I_{CaL} (89% HDPI)	I_{to} (89% HDPI)	I_{Kur} (89% HDPI)
N	RP (mV)	-74.1	-73.7 (-76.3, -17.5)	-72.7 (-72.7, -72.5)	-62.2 (-63.0, -61.4)
	AMP (mV)	114.3	113.4 (39.0, 115.8)	113.4 (113.1, 113.6)	95.0 (94.2, 95.8)
	APD90 (ms)	223.2	217.5 (16, 295)	271 (266, 280)	298 (295, 302)
C	RP (mV)	-81.2	-81.7 (-81.8, -81.6)	-81.3 (-81.4, -81.2)	-81.6 (-81.8, -80.9)
	AMP (mV)	110.9	112.5 (112.3, 112.9)	109.4 (107.5, 110.9)	111.2 (109.8, 111.8)
	APD90 (ms)	288.5	211 (189, 246)	257 (247, 266)	237.5 (190, 304)
N+S	RP (mV)	-74.1	-76.3 (-76.7, -70.3)	-73.2 (-73.2, -73.1)	-62.2 (-66.1, -58.0)
	AMP (mV)	114.3	118.6 (106.1, 120.3)	110.8 (110.6, 111.0)	93.9 (88.2, 98.8)
	APD90 (ms)	223.2	172.5 (131, 233)	248 (245, 249)	286 (283, 293)
C+S	RP (mV)	-81.2	-81.5 (-82.0, -27.1)	-81.2 (-81.2, -81.2)	-81.9 (-82.0, -81.8)
	AMP (mV)	110.9	111.7 (39.4, 112.9)	109.2 (109.1, 109.3)	107.6 (106.4, 108.9)
	APD90 (ms)	288.5	192 (35, ∞)	267 (264, 270)	179.5 (150, 206)

Supplementary Table 2: Effect on full AP of using parameter posterior distributions to calibrate channel models. Measurements taken are resting potential (RP), action potential amplitude (AMP) and action potential duration to 90% repolarisation (APD90). N: Nygren model, C: Courtemanche model, +S: represents the indicated channel was replaced with the standardised form in the full AP model.

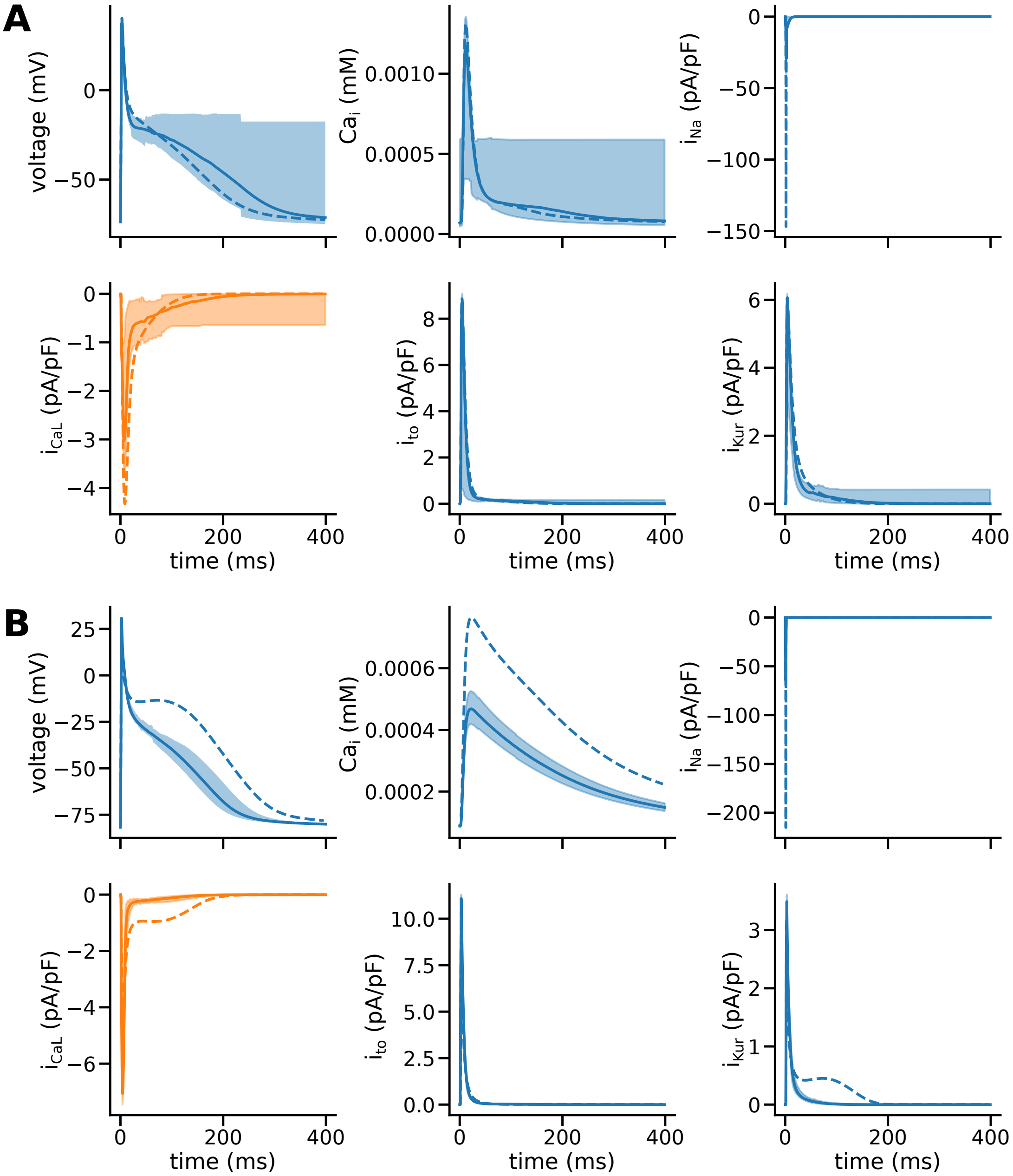

Supplementary Figure 13: Action potential, major currents and intracellular calcium concentrations for A: N model and B: C model. Traces using published parameters are represented with dashed lines. Samples from the full cell model with I_{CaL} replaced by the unified recalibrated parameterisation are displayed as a median line with 89% high density posterior intervals. The orange plot highlights the channel that is recalibrated using the parameter posterior distribution from ABC. All other model parameters are unchanged from published values.

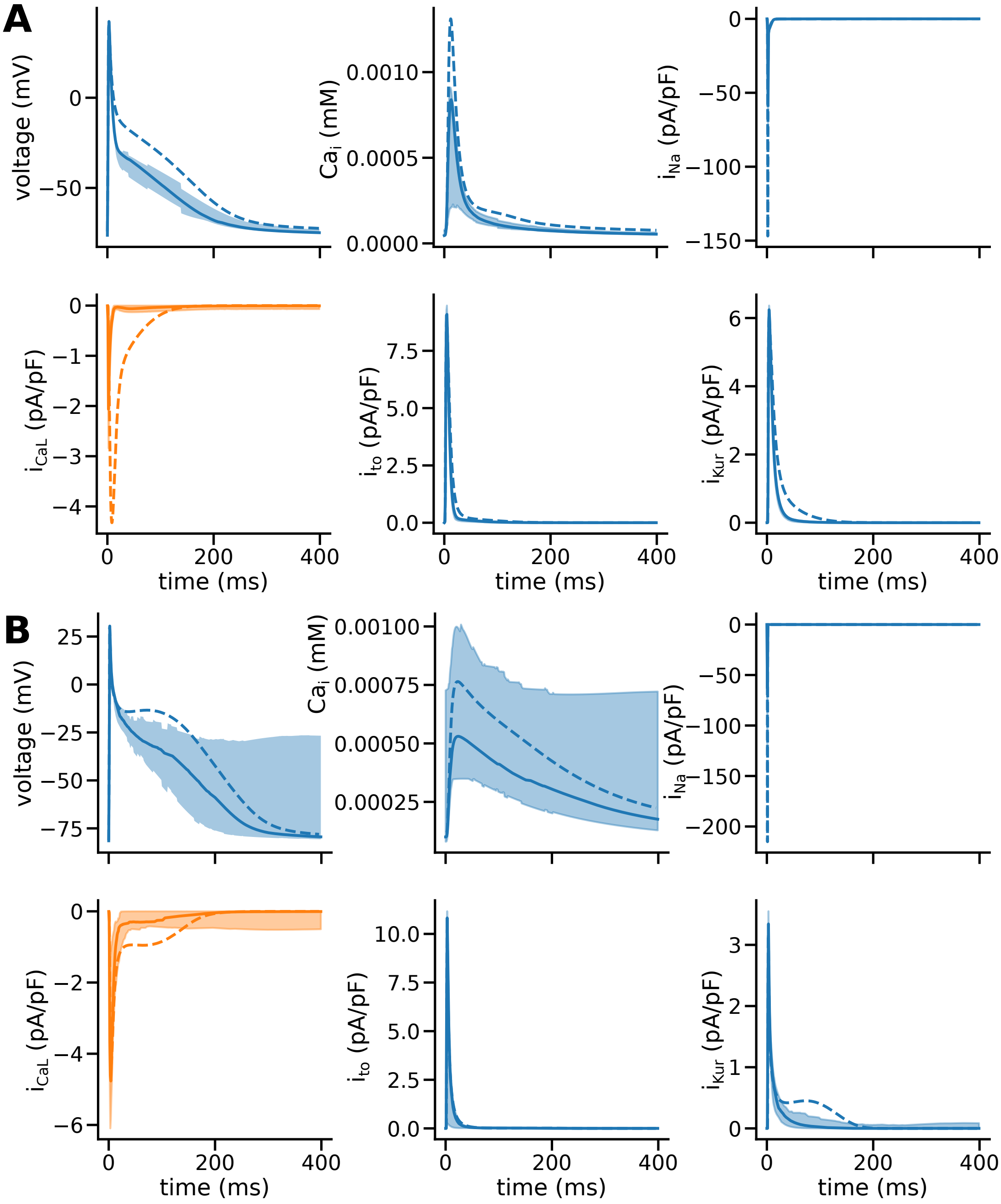

Supplementary Figure 14: Action potential, major currents and intracellular calcium concentrations for A: N model and B: C model, with the S form of I_{CaL} replacing the original form. Traces using published parameters are represented with dashed lines. Samples from the full cell model with I_{CaL} replaced by the unified recalibrated parameterisation are displayed as a median line with 89% high density posterior intervals. The orange plot highlights the channel that is replaced with the standardised model and using the parameter posterior distribution from ABC. All other model parameters are unchanged from published values.

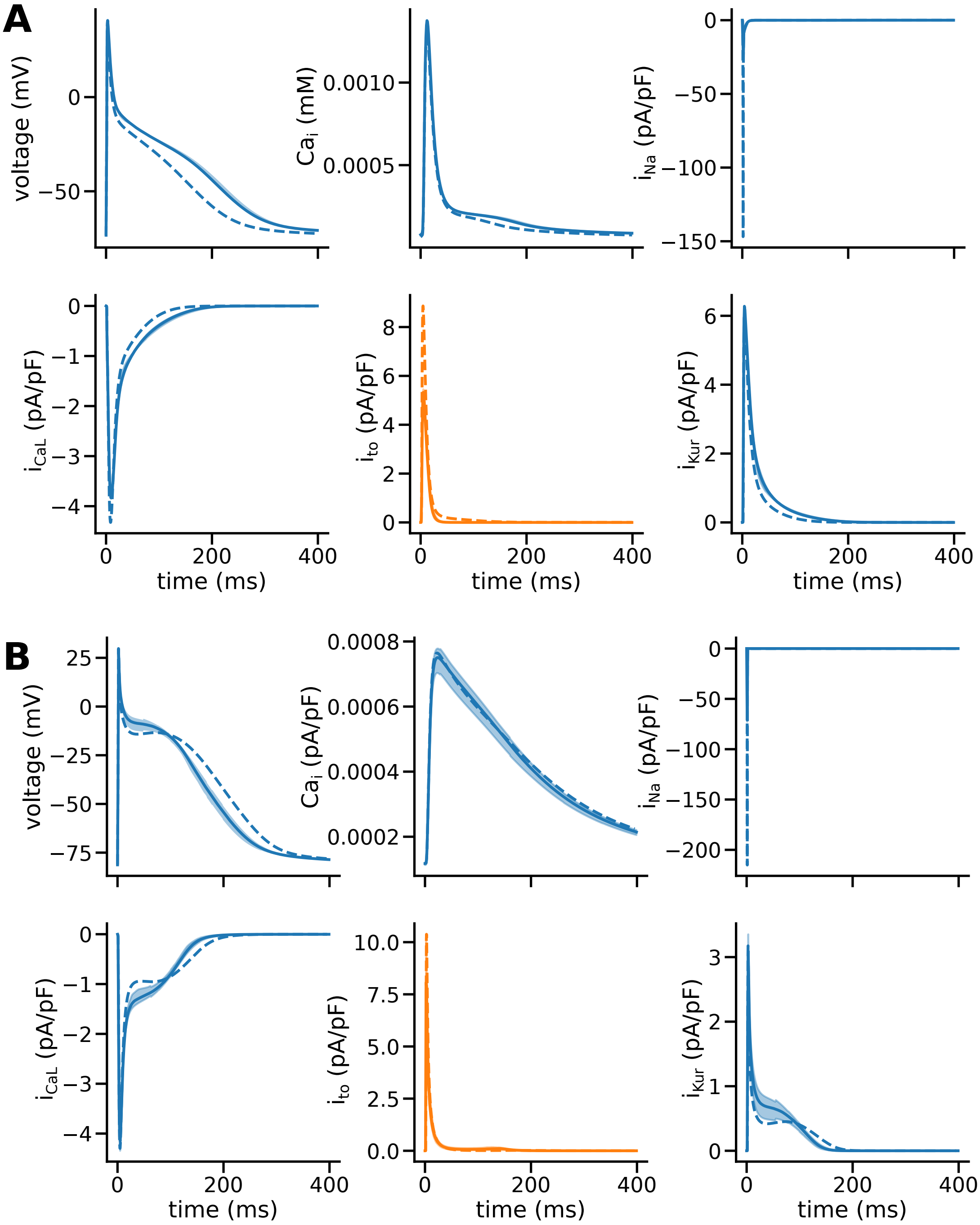

Supplementary Figure 15: Action potential, major currents and intracellular calcium concentrations for A: N model and B: C model. Traces using published parameters are represented with dashed lines. Samples from the full cell model with I_{to} replaced by the unified recalibrated parameterisation are displayed as a median line with 89% high density posterior intervals.. The orange plot highlights the channel that is recalibrated using the parameter posterior distribution from ABC. All other model parameters are unchanged from published values.

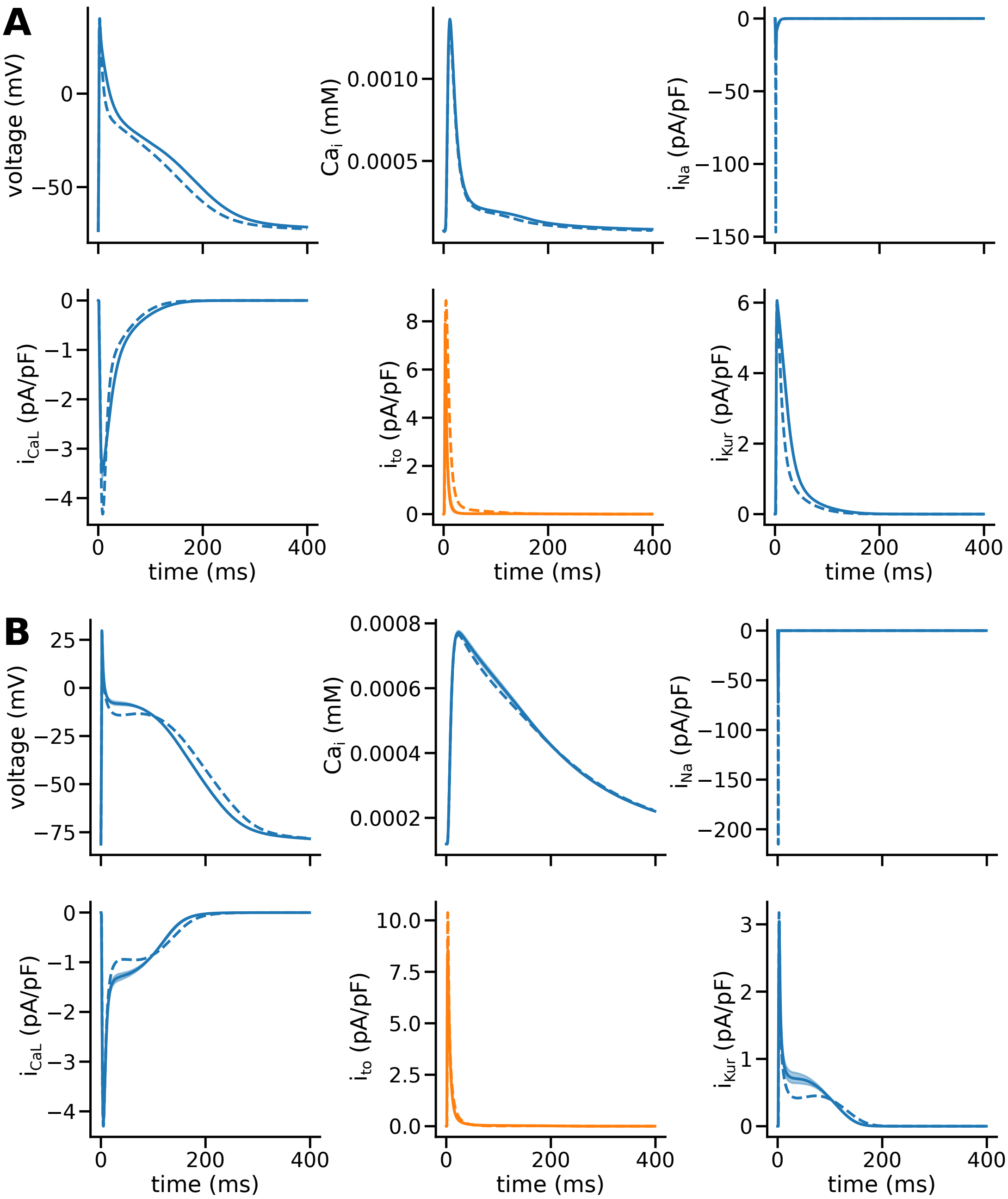

Supplementary Figure 16: Action potential, major currents and intracellular calcium concentrations for A: N model and B: C model, , with the S form of I_{to} replacing the original form. Traces using published parameters are represented with dashed lines. Samples from the full cell model with I_{to} replaced by the unified recalibrated parameterisation are displayed as a median line with 89% high density posterior intervals. The orange plot highlights the channel that is replaced with the standardised model and using the parameter posterior distribution from ABC. All other model parameters are unchanged from published values.

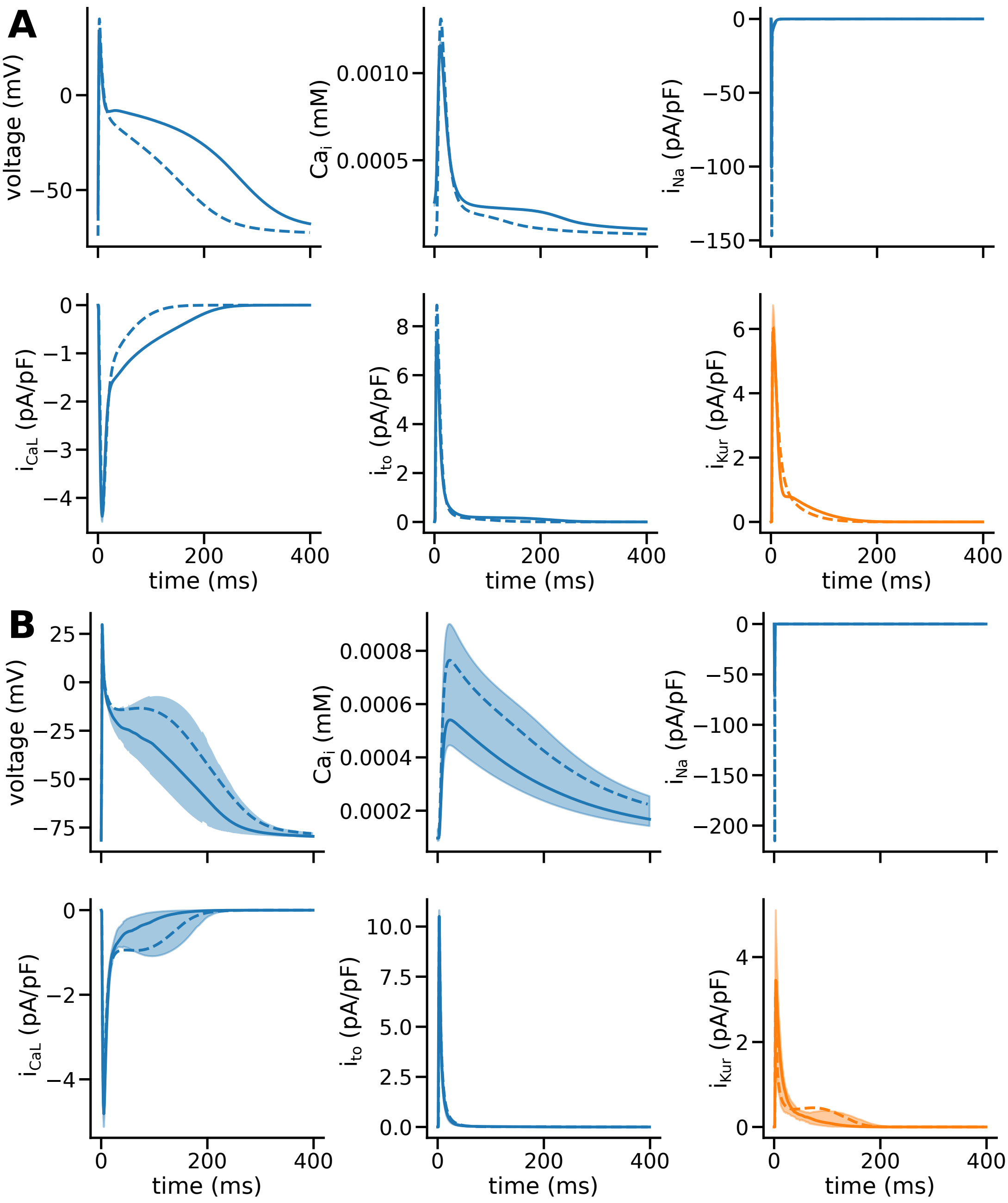

Supplementary Figure 17: Action potential, major currents and intracellular calcium concentrations for A: N model and B: C model. Traces using published parameters are represented with dashed lines. Samples from the full cell model with I_{Kur} replaced by the unified recalibrated parameterisation are displayed as a median line with 89% high density posterior intervals. The orange plot highlights the channel that is recalibrated using the parameter posterior distribution from ABC. All other model parameters are unchanged from published values.

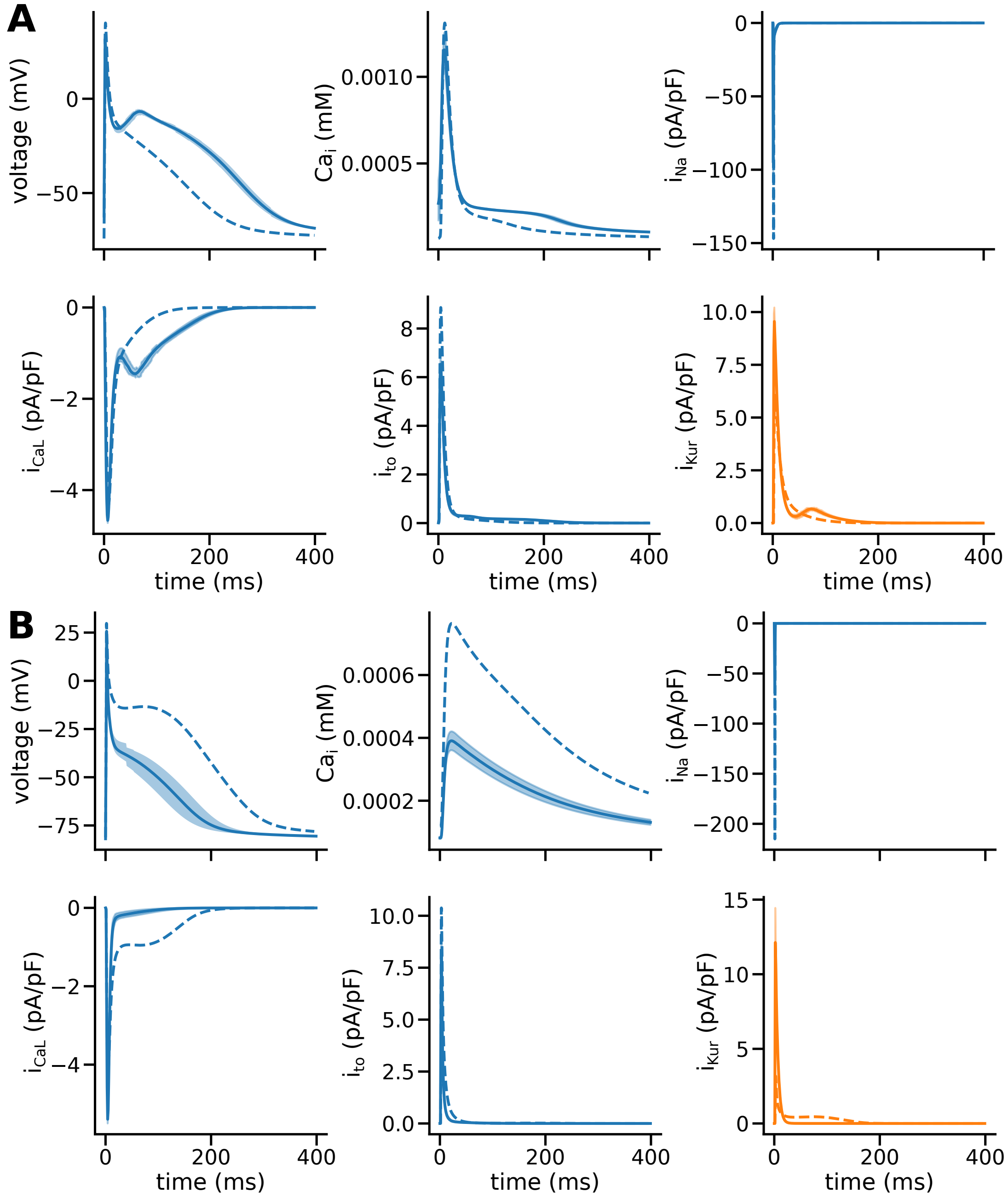

Supplementary Figure 18: Action potential, major currents and intracellular calcium concentrations for A: N model and B: C model, , with the S form of I_{Kur} replacing the original form. Traces using published parameters are represented with dashed lines. Samples from the full cell model with I_{Kur} replaced by the unified recalibrated parameterisation are displayed as a median line with 89% high density posterior intervals. The orange plot highlights the channel that is replaced with the standardised model and using the parameter posterior distribution from ABC. All other model parameters are unchanged from published values.

S4 Model equations and numerical results

S4.1 I_{Na}

$$I_{\mathrm{Na}} = P_{\mathrm{Na}} m^3 \left(\mathbf{s_1} h_1 + (1-\mathbf{s_1}) h_2\right) \left[\mathrm{Na}^+\right]_{\mathrm{c}} \mathrm{V} \frac{\mathrm{F}^2}{\mathrm{RT}} \frac{e^{(\mathrm{V}-\mathrm{E_{Na}})\mathrm{F/RT}} - 1.0}{e^{\mathrm{VF/RT}} - 1.0}$$

$$\frac{\mathrm{d}m}{\mathrm{dt}} = \frac{\overline{m} - m}{\tau_m}, \qquad \frac{\mathrm{d}h_1}{\mathrm{dt}} = \frac{\overline{h} - h_1}{\tau_{h_1}}, \qquad \frac{\mathrm{d}h_2}{\mathrm{dt}} = \frac{\overline{h} - h_2}{\tau_{h_2}}$$

$$\overline{m} = \frac{1.0}{1.0 + e^{-(\mathrm{V}+\mathbf{r_1})/\mathbf{r_2}}}, \qquad \tau_m = 1000(\mathbf{r_3} e^{-((\mathrm{V}+\mathbf{r_4})/\mathbf{r_5})^2} + \mathbf{r_6})$$

$$\overline{h} = \frac{1.0}{1.0 + e^{(\mathrm{V}+\mathbf{q_1})/\mathbf{q_2}}}$$

$$\tau_{h_1} = 1000\left(\frac{\mathbf{q_3}}{1.0 + e^{(\mathrm{V}+\mathbf{q_4})/\mathbf{q_5}}} + \mathbf{q_6}\right), \qquad \tau_{h_2} = 1000\left(\frac{\mathbf{q_7}}{1.0 + e^{(\mathrm{V}+\mathbf{q_4})/\mathbf{q_5}}} + \mathbf{q_8}\right)$$

Supplementary Table 3: Gating kinetics in Nygren model of I_{Na} channel current(see Table 6 in [1]). Time constants are multiplied by 1000 to convert from s to ms.

			Original			Unified		
Name	Published	Prior	Median	89% HDPI	$\log_{10}$RSD	Median	89% HDPI	$\log_{10}$RSD
r1	27.12	$\mathcal{U}$(0, 100)	52.25	(50.93, 53.52)	-1.795	42.83	(33.91, 55.13)	-0.7966
r2	8.21	$\mathcal{U}$(0, 20)	10.69	(9.769, 11.64)	-1.256	13.19	(7.761, 19.94)	-0.5127
r3*	4.2e-05	$\mathcal{U}$(-6, -3)	2.097e-05	(1.009e-06, 0.000325)	-0.749	1.371e-05	(1.002e-06, 0.0002247)	-0.7856
r4	25.57	$\mathcal{U}$(0, 100)	57.99	(22.82, 99.97)	-0.3404	56.79	(16.97, 99.79)	-0.293
r5	28.8	$\mathcal{U}$(0, 20)	9.979	(0.2856, 17.75)	-0.2282	8.932	(0.03437, 17.49)	-0.1981
r6*	2.4e-05	$\mathcal{U}$(-6, -3)	2.191e-05	(1.001e-06, 0.0005717)	-0.7021	9.662e-05	(5.195e-05, 0.0001536)	-1.43
q1	63.6	$\mathcal{U}$(0, 200)	95.57	(95.11, 96.11)	-2.465	93.94	(88.3, 99.55)	-1.4
q2	5.3	$\mathcal{U}$(0, 20)	6.5	(6.085, 6.841)	-1.432	8.182	(1.222, 13.13)	-0.3487
q3*	0.03	$\mathcal{U}$(-3, 0)	0.09214	(0.001002, 0.4502)	-0.1696	0.004096	(0.002543, 0.02547)	-0.7312
q4	35.1	$\mathcal{U}$(0, 100)	34.98	(0.1474, 147.7)	-0.02249	53.57	(48.79, 57.53)	-1.263
q5	3.2	$\mathcal{U}$(0, 20)	12.76	(4.548, 19.99)	-0.361	10.61	(7.184, 13.18)	-0.7463
q6*	0.0003	$\mathcal{U}$(-5, -2)	0.0002548	(1.002e-05, 0.004472)	-0.6137	0.0002403	(1.006e-05, 0.001556)	-0.7811
q7*	0.12	$\mathcal{U}$(-3, 0)	0.07443	(0.001005, 0.3999)	-0.1983	0.02022	(0.002551, 0.02706)	-0.6835
q8*	0.003	$\mathcal{U}$(-4, -1)	0.002224	(0.0001001, 0.04665)	-0.4589	0.0005268	(0.0001001, 0.00171)	-0.9219
s1	0.9	$\mathcal{U}$(0, 1)	0.5015	(0.08985, 0.9754)	-0.2317	0.4717	(0.1367, 0.8259)	-0.334

Supplementary Table 4: Summary of results for parameters of Nygren I_{Na} model using original and unified dataset. *Parameters were searched in $\log_{10}$ space based on the scale of their published values and are presented in linear space. The prior for these parameters is still in the original $\log_{10}$ space.

$$I_{\mathrm{Na}} = g_{\mathrm{Na}} m^3 h j(\mathrm{V} - \mathrm{E}_{\mathrm{Na}})$$

$$\frac{\mathrm{d}\phi}{\mathrm{dt}} = \frac{\phi_\infty - \phi}{\tau_\phi}, \qquad \text{for } \phi = m, h, j$$

$$\tau_\phi = (\alpha_\phi + \beta_\phi)^{-1}, \qquad \phi_\infty = \alpha_\phi \tau_\phi, \qquad \text{for } \phi = m, h, j$$

$$\alpha_m = \begin{cases} \mathbf{a_{m,2}} \frac{\mathrm{V} - \mathbf{a_{m,1}}}{1 - e^{-\mathbf{a_{m,3}}(\mathrm{V} - \mathbf{a_{m,1}})}} \\ \mathbf{a_{m,4}}, \text{ if } \mathrm{V} = \mathbf{a_{m,1}} \end{cases}, \qquad \beta_m = \mathbf{b_{m,1}} e^{-\mathrm{V}/\mathbf{b_{m,2}}}$$

$$\alpha_h = \begin{cases} \mathbf{a_{h,1}} e^{(\mathrm{V} + \mathbf{a_{h,3}})/-\mathbf{a_{h,2}}} \\ a_{h,5}, \qquad \text{if } \mathrm{V} \geq \mathbf{c_{h,1}} \end{cases}, \qquad a_{h,5} = \mathbf{a_{h,1}} e^{(\mathbf{c_{h,1}} + \mathbf{a_{h,3}})/-\mathbf{a_{h,2}}}$$

$$\beta_h = \begin{cases} \mathbf{b_{h,4}} e^{\mathbf{b_{h,5}}\mathrm{V}} + \mathbf{b_{h,6}} e^{\mathbf{b_{h,7}}\mathrm{V}} \\ \left(b_{h,1} \left[1.0 + e^{(V + \mathbf{b_{h,2}})/-\mathbf{b_{h,3}}}\right]\right)^{-1}, \quad \text{if } V \geq \mathbf{c_{h,1}} \end{cases}$$

$$b_{h,1} = \left(\mathbf{b_{h,4}} e^{\mathbf{b_{h,5}} \mathbf{c_{h,1}}} + \mathbf{b_{h,6}} e^{\mathbf{b_{h,7}} \mathbf{c_{h,1}}}\right)^{-1} \left(1.0 + e^{(\mathbf{c_{h,1}} + \mathbf{b_{h,2}})/-\mathbf{b_{h,3}}}\right)^{-1}$$

$$\alpha_j = \begin{cases} \left[-\mathbf{a_{j,1}} e^{\mathbf{a_{j,2}}\mathrm{V}} - \mathbf{a_{j,3}} e^{-\mathbf{a_{j,4}}\mathrm{V}}\right] \frac{\mathrm{V} + a_{j,5}}{1.0 + e^{\mathbf{a_{j,6}}(\mathrm{V} + \mathbf{a_{j,7}})}} \\ 0, \quad \text{if } V \geq \mathbf{c_{j,1}} \end{cases}, \qquad a_{j,5} = -\mathbf{c_{j,1}}$$

$$\beta_j = \begin{cases} \mathbf{b_{j,5}} \frac{e^{-\mathbf{b_{j,6}}\mathrm{V}}}{1 + e^{-\mathbf{b_{j,7}}(\mathrm{V} + \mathbf{b_{j,8}})}} \\ b_{j,1} \frac{e^{-b_{j,2}\mathrm{V}}}{1 + e^{-\mathbf{b_{j,3}}(\mathrm{V} + \mathbf{b_{j,4}})}}, \quad \text{if } V \geq \mathbf{c_{j,1}} \end{cases},$$

$$b_{j,1} = \mathbf{b_{j,5}} \frac{e^{-\mathbf{b_{j,6}} \mathbf{c_{j,1}}}}{1 + e^{-\mathbf{b_{j,7}}(\mathbf{c_{j,1}} + \mathbf{b_{j,8}})}} \left[\frac{e^{-b2_j \mathbf{c_{j,1}}}}{(1 + e^{-\mathbf{b_{j,3}}(\mathbf{c_{j,1}} + \mathbf{b_{j,4}})}}\right]^{-1}, \qquad b_{j,2} = 0.0$$

Supplementary Table 5: Gating kinetics in Courtemanche model of I_{Na} channel current (see Appendix in [2]). Values of $a_{h,5}$, $b_{h,1}$, $a_{j,5}$ and $b_{j,1}$ are set to enforce continuity in piecewise functions. $b_{j,2}$ is set to zero to reduce dimensionality of the calibration problem as the published parameter was effectively zero (2.535×10^{-7}).

Original/Unified					
Name	Published	Prior	Median	89% HDPI	$\log_{10}$RSD
$a1_m$	-47.13	$\mathcal{U}(-100, 0)$	-75.97	(-79.77, -73.65)	-1.586
$a2_m$	0.32	$\mathcal{U}(0, 1)$	0.2639	(0.2476, 0.2741)	-1.476
$a3_m$	0.1	$\mathcal{U}(0, 1)$	0.3355	(0.1947, 0.7643)	-0.2765
$a4_m$	3.2	$\mathcal{U}(0, 10)$	4.895	(1.699, 9.998)	-0.2421
$b1_m$	0.08	$\mathcal{U}(0, 10)$	1.263	(1.225, 1.322)	-1.64
$b2_m$	11.0	$\mathcal{U}(0, 100)$	23.21	(22.48, 23.74)	-1.774
$a1_h^*$	0.135	$\mathcal{U}(-2, 1)$	0.3845	(0.01157, 3.35)	0.269
$a2_h$	6.8	$\mathcal{U}(0, 50)$	15.48	(14.49, 15.99)	-1.465
$a3_h$	80.0	$\mathcal{U}(0, 200)$	122.2	(67.51, 155.4)	-0.6085
$b2_h$	10.66	$\mathcal{U}(0, 100)$	18.35	(5.525, 31.86)	-0.3218
$b3_h$	11.1	$\mathcal{U}(0, 50)$	16.73	(12.42, 21.47)	-0.7582
$b4_h^*$	3.56	$\mathcal{U}(-1, 2)$	15.11	(14.51, 15.79)	-1.999
$b5_h^*$	0.079	$\mathcal{U}(-3, 0)$	0.05808	(0.05716, 0.05887)	-2.492
$b6_h^*$	310000.0	$\mathcal{U}(3, 6)$	21500.0	(1002.0, 321300.0)	-0.6777
$b7_h^*$	0.35	$\mathcal{U}(-2, 1)$	1.327	(0.2776, 6.324)	0.4741
$c1_h$	-40.0	$\mathcal{U}(-100, 0)$	-37.27	(-45.94, -32.91)	-0.8902
$a1_j^*$	127100.0	$\mathcal{U}(3, 7)$	123700.0	(1180.0, 4778000.0)	-0.6361
$a2_j^*$	0.2444	$\mathcal{U}(-2, 2)$	5.144	(0.1528, 50.75)	0.02476
$a3_j^*$	3.474e-05	$\mathcal{U}(-5, -1)$	0.0003798	(1.015e-05, 0.001191)	-0.7783
$a4_j^*$	0.04391	$\mathcal{U}(-4, 0)$	0.003784	(0.0001, 0.02896)	-0.5131
$a6_j$	0.311	$\mathcal{U}(0, 1)$	0.4461	(0.01244, 0.8846)	-0.2087
$a7_j$	79.23	$\mathcal{U}(0, 100)$	41.53	(4.671, 91.36)	-0.1966
$b3_j$	0.1	$\mathcal{U}(0, 1)$	0.4481	(0.001746, 0.8611)	-0.1977
$b4_j$	32.0	$\mathcal{U}(0, 100)$	67.64	(21.51, 99.97)	-0.3641
$b5_j$	0.1212	$\mathcal{U}(0, 1)$	0.3552	(0.04405, 0.7968)	-0.1691
$b6_j^*$	0.01052	$\mathcal{U}(-4, 0)$	0.001531	(0.0001004, 0.01829)	-0.5618
$b7_j$	0.1378	$\mathcal{U}(0, 1)$	0.2679	(0.0008117, 0.7773)	-0.09422
$b8_j$	40.14	$\mathcal{U}(0, 100)$	72.38	(31.39, 99.94)	-0.4359
$c1_j$	-40.0	$\mathcal{U}(-100, 0)$	-33.07	(-74.3, -0.5147)	-0.179

Supplementary Table 6: Summary of results for parameters of Courtemanche I_{Na} model using original/unified (equivalent) dataset. *Parameters were searched in $\log_{10}$ space based on the scale of their published values and are presented in linear space. The prior for these parameters is still in the original $\log_{10}$ space.

Unified				
Name	Prior	Median	89% HDPI	$\log_{10}$RSD
p_1^*	$\mathcal{U}$(1, 5)	5134.0	(1620.0, 9786.0)	-1.241
p_2	$\mathcal{U}$(1e-7, 0.2)	0.126	(0.1048, 0.1524)	-0.9488
p_3^*	$\mathcal{U}$(-3, 1)	3.374	(0.5586, 6.144)	-0.2008
p_4	$\mathcal{U}$(1e-7, 0.4)	0.0234	(0.002225, 0.04426)	-0.2237
p_5^*	$\mathcal{U}$(-1, 3)	6.091	(5.572, 6.662)	-1.495
p_6	$\mathcal{U}$(1e-7, 0.2)	0.0441	(0.04209, 0.04601)	-1.523
p_7^*	$\mathcal{U}$(-4, 0)	0.005353	(0.004147, 0.006667)	-1.496
p_8	$\mathcal{U}$(1e-7, 0.2)	0.04053	(0.03871, 0.04302)	-1.44
A^*	$\mathcal{U}$(0, 1)	4.195	(4.049, 4.314)	-1.869

Supplementary Table 7: Summary of results for parameters of standardised I_{Na} model using unified dataset. *Parameters were searched in $\log_{10}$ space and are presented in linear space. The prior for these parameters is still in the original $\log_{10}$ space.

S4.2 I_{CaL}

$$
\begin{aligned}
I_{\mathrm{CaL}} &= \bar{g}_{\mathrm{Ca,L}} d_{\mathrm{L}} \left[f_{\mathrm{Ca}} f_{\mathrm{L}_1} + (1 - f_{\mathrm{Ca}}) f_{\mathrm{L}_2} \right] (\mathrm{V} - \mathrm{E}_{\mathrm{Ca,app}}) \\
\frac{\mathrm{d}d_{\mathrm{L}}}{\mathrm{dt}} &= \frac{\overline{d_{\mathrm{L}}} - d_{\mathrm{L}}}{\tau_{d_{\mathrm{L}}}}, \qquad \frac{\mathrm{d}f_{\mathrm{L}_1}}{\mathrm{dt}} = \frac{\overline{f_{\mathrm{L}}} - f_{\mathrm{L}_1}}{\tau_{f_{\mathrm{L}_1}}}, \qquad \frac{\mathrm{d}f_{\mathrm{L}_2}}{\mathrm{dt}} = \frac{\overline{f_{\mathrm{L}}} - f_{\mathrm{L}_2}}{\tau_{f_{\mathrm{L}_2}}} \\
\overline{d_{\mathrm{L}}} &= \frac{1.0}{1.0 + e^{(\mathrm{V}+\mathbf{p_1})/-\mathbf{p_2}}}, \qquad \tau_{d_{\mathrm{L}}} = 1000 \left(\mathbf{p_3} e^{-((\mathrm{V}+\mathbf{p_4})/\mathbf{p_5})^2} + \mathbf{p_6} \right) \\
\overline{f_{\mathrm{L}}} &= \frac{1.0}{1.0 + e^{(\mathrm{V}+\mathbf{q_1})/\mathbf{q_2}}} \\
\tau_{f_{\mathrm{L}_1}} &= 1000 \left(\mathbf{q_3} e^{-((\mathrm{V}+\mathbf{q_4})/\mathbf{q_5})^2} + \mathbf{q_6} \right), \qquad \tau_{f_{\mathrm{L}_2}} = 1000 \left(\mathbf{r_1} e^{-((\mathrm{V}+\mathbf{r_2})/\mathbf{r_3})^2} + \mathbf{r_4} \right)
\end{aligned}
$$

Supplementary Table 8: Gating kinetics in Nygren model of I_{CaL} channel current (see Table 7 in [1]). Time constants are multiplied by 1000 to convert from s to ms.

			Original			Unified		
Name	Published	Prior	Median	89% HDPI	$\log_{10}$RSD	Median	89% HDPI	$\log_{10}$RSD
p1	9.0	$\mathcal{U}$(-100, 100)	2.684	(2.529, 2.906)	-1.29	9.317	(5.103, 10.51)	-0.5683
p2	5.8	$\mathcal{U}$(0, 50)	5.055	(5.022, 5.083)	-2.416	7.378	(6.679, 8.916)	-0.9407
p3*	0.0027	$\mathcal{U}$(-7, 3)	0.1048	(0.096, 0.1063)	-1.869	0.001129	(1e-07, 0.02533)	-0.2353
p4	35.0	$\mathcal{U}$(-100, 100)	-11.51	(-11.6, -11.36)	-2.151	-39.18	(-99.98, 74.02)	0.4508
p5	30.0	$\mathcal{U}$(0, 50)	18.58	(18.39, 18.86)	-2.078	28.21	(6.568, 49.05)	-0.2817
p6*	0.002	$\mathcal{U}$(-7, 3)	0.00996	(0.009237, 0.0108)	-1.919	0.0009303	(1.027e-07, 0.0009643)	-0.5757
q1	27.4	$\mathcal{U}$(0, 100)	32.02	(21.21, 47.51)	-0.5415	32.02	(21.21, 47.51)	-0.5415
q2	7.1	$\mathcal{U}$(0, 50)	8.06	(1.553, 18.14)	-0.2068	8.06	(1.553, 18.14)	-0.2068
q3*	0.161	$\mathcal{U}$(-7, 3)	0.04208	(1.103e-07, 0.7733)	-0.002009	0.04208	(1.103e-07, 0.7733)	-0.002009
q4	40.0	$\mathcal{U}$(0, 100)	59.81	(29.52, 99.4)	-0.3934	59.81	(29.52, 99.4)	-0.3934
q5	14.4	$\mathcal{U}$(0, 50)	25.73	(2.594, 43.97)	-0.2737	25.73	(2.594, 43.97)	-0.2737
q6*	0.01	$\mathcal{U}$(-7, 3)	0.007372	(0.002444, 0.05932)	-0.5916	0.007372	(0.002444, 0.05932)	-0.5916
r1*	1.332	$\mathcal{U}$(-7, 3)	0.05255	(1.14e-07, 2.713)	0.05333	0.05255	(1.14e-07, 2.713)	0.05333
r2	40.0	$\mathcal{U}$(0, 100)	45.95	(13.92, 96.56)	-0.2224	45.95	(13.92, 96.56)	-0.2224
r3	14.2	$\mathcal{U}$(0, 100)	46.89	(0.5929, 88.34)	-0.1519	46.89	(0.5929, 88.34)	-0.1519
r4*	0.0626	$\mathcal{U}$(-7, 3)	0.01455	(1.051e-07, 0.05889)	-0.2101	0.01455	(1.051e-07, 0.05889)	-0.2101

Supplementary Table 9: Summary of results for parameters of Nygren I_{CaL} model. Only activation parameters (p) recalibrated to unified dataset. *Parameters were searched in $\log_{10}$ space based on the scale of their published values and are presented in linear space. The prior for these parameters is still in the original $\log_{10}$ space.

$$
\begin{aligned}
I_{\mathrm{CaL}} &= g_{\mathrm{Ca,L}} d f f_{\mathrm{Ca}} (\mathrm{V} - \mathrm{E}_{\mathrm{Ca,app}}) \\
\frac{\mathrm{d}d}{\mathrm{dt}} &= \frac{\overline{d} - d}{\tau_d}, \qquad \frac{\mathrm{d}f}{\mathrm{dt}} = \frac{\overline{f} - f}{\tau_f} \\
\overline{d} &= \frac{1.0}{1.0 + e^{(\mathrm{V}+\mathbf{p_4})/-\mathbf{p_5}}}, \qquad \tau_d = \frac{1 - e^{(\mathrm{V}+\mathbf{p_1})/-\mathbf{p_2}}}{\mathbf{p_3}(\mathrm{V} + \mathbf{p_1}) \left[1 + e^{(\mathrm{V}+\mathbf{p_1})/-\mathbf{p_2}}\right]} \\
\overline{f} &= \frac{1.0}{1.0 + e^{(\mathrm{V}+\mathbf{q_6})/\mathbf{q_7}}}, \qquad \tau_f = \mathbf{q_1} \left[\mathbf{q_2} e^{-\mathbf{q_3}^2 (\mathrm{V}+\mathbf{q_4})^2} + \mathbf{q_5}\right]^{-1}
\end{aligned}
$$

Supplementary Table 10: Gating kinetics in Courtemanche model of I_{CaL} channel current (see Appendix in [2]).

			Original			Unified		
Name	Published	Prior	Median	89% HDPI	$\log_{10}$RSD	Median	89% HDPI	$\log_{10}$RSD
p1	10.0	$\mathcal{U}$(-100, 100)	-63.91	(-90.59, -30.08)	-0.5153	-38.24	(-99.15, 54.23)	0.2831
p2	6.24	$\mathcal{U}$(0, 50)	9.655	(1.853, 18.57)	-0.2793	19.17	(0.1832, 43.09)	-0.1334
p3*	0.035	$\mathcal{U}$(-7, 3)	2.072e-07	(1.559e-07, 0.0001709)	-0.6749	0.02479	(0.01392, 0.0721)	-0.7863
p4	10.0	$\mathcal{U}$(-100, 100)	19.07	(6.624, 20.71)	-0.5022	10.44	(10.05, 10.71)	-1.66
p5	8.0	$\mathcal{U}$(0, 50)	6.078	(5.647, 7.053)	-1.029	7.447	(7.267, 7.638)	-1.754
q1*	9.0	$\mathcal{U}$(0, 3)	57.97	(1.072, 435.5)	-0.372	5.315	(1.004, 19.81)	-0.2447
q2*	0.0197	$\mathcal{U}$(-2, 3)	0.2487	(0.01006, 3.008)	0.2592	1.825	(0.1754, 6.422)	0.3113
q3*	0.0337	$\mathcal{U}$(-4, 0)	0.01259	(0.0001002, 0.1925)	-0.3061	0.1147	(0.08051, 0.1534)	-1.006
q4	10.0	$\mathcal{U}$(-100, 100)	-11.13	(-71.3, 88.69)	0.8784	1.305	(-1.347, 4.125)	0.07887
q5*	0.02	$\mathcal{U}$(-4, 0)	0.02488	(0.0001023, 0.4122)	-0.1906	0.1955	(0.03626, 0.6931)	-0.2331
q6	28.0	$\mathcal{U}$(-100, 100)	27.82	(25.28, 30.52)	-1.177	33.32	(25.89, 40.27)	-0.8614
q7	6.9	$\mathcal{U}$(0, 50)	6.879	(5.583, 8.993)	-0.8052	10.95	(7.257, 13.98)	-0.704

Supplementary Table 11: Summary of results of parameters of Courtemanche I_{CaL} model. *Parameters were searched in $\log_{10}$ space based on the scale of their published values and are presented in linear space. The prior for these parameters is still in the original $\log_{10}$ space.

Unified				
Name	Prior	Median	89% HDPI	$\log_{10}$RSD
p_1^*	$\mathcal{U}$(-7, 3)	0.5513	(1.02e-07, 1.073)	0.1053
p_2	$\mathcal{U}$(1e-7, 0.4)	0.01878	(1.245e-05, 0.05856)	-0.07143
p_3^*	$\mathcal{U}$(-7, 3)	0.375	(0.0056, 1.575)	0.07778
p_4	$\mathcal{U}$(1e-7, 0.4)	0.09998	(0.02278, 0.2473)	-0.1811
p_5^*	$\mathcal{U}$(-7, 3)	0.06489	(0.04241, 0.2347)	-0.6597
p_6	$\mathcal{U}$(1e-7, 0.4)	0.04437	(0.03053, 0.06118)	-0.6387
p_7^*	$\mathcal{U}$(-7, 3)	0.02056	(0.01379, 0.02842)	-1.205
p_8	$\mathcal{U}$(1e-7, 0.4)	0.02449	(0.01303, 0.03353)	-0.5575
A^*	$\mathcal{U}$(0, 3)	4.497	(3.235, 13.56)	-0.5354

Supplementary Table 12: Summary of results of parameters of standardised I_{CaL} model using unified dataset. *Parameters were searched in $\log_{10}$ space and are presented in linear space. The prior for these parameters is still in the original $\log_{10}$ space.

S4.3 I_{to}

$$I_{\mathrm{to}} = \bar{g}_{\mathrm{t}} r s \left(\mathrm{V} - \mathrm{E_K}\right)$$

$$\frac{\mathrm{d}r}{\mathrm{dt}} = \frac{\overline{r} - r}{\tau_r}, \qquad \frac{\mathrm{d}s}{\mathrm{dt}} = \frac{\overline{s} - s}{\tau_s}$$

$$\overline{r} = \frac{1.0}{1.0 + e^{(\mathrm{V} - \mathbf{p_1})/-\mathbf{p_2}}}, \qquad \tau_r = 1000\left(\mathbf{p_3} e^{-(\mathrm{V}/\mathbf{p_4})^2} + \mathbf{p_5}\right)$$

$$\overline{s} = \frac{1.0}{1.0 + e^{(\mathrm{V} + \mathbf{q_1})/\mathbf{q_2}}}, \qquad \tau_s = 1000\left(\mathbf{q_3} e^{-((\mathrm{V} + \mathbf{q_4})/\mathbf{q_5})^2} + \mathbf{q_6}\right)$$

Supplementary Table 13: Gating kinetics in Nygren model of I_{to} channel current (see Table 8 in [1]). Time constants are multiplied by 1000 to convert from s to ms.

			Original			Unified		
Name	Published	Prior	Median	89% HDPI	$\log_{10}$RSD	Median	89% HDPI	$\log_{10}$RSD
p1	1.0	$\mathcal{U}$(-100, 100)	3.25	(0.3302, 6.375)	-0.234	-0.577	(-7.205, 2.269)	0.4008
p2	11.0	$\mathcal{U}$(1e-7, 50)	10.32	(7.678, 12.61)	-0.8453	2.197	(1.381, 2.753)	-0.6039
p3*	0.0035	$\mathcal{U}$(-7, 0)	4.613e-06	(1.013e-07, 0.0001495)	-0.6929	0.008113	(0.007376, 0.008801)	-1.931
p4	30.0	$\mathcal{U}$(1e-7, 50)	22.81	(0.2115, 43.89)	-0.2055	43.54	(40.81, 46.05)	-1.426
p5*	0.0015	$\mathcal{U}$(-7, 0)	0.002083	(1.005e-07, 0.004469)	-0.4898	0.001244	(0.00109, 0.00137)	-1.973
q1	40.5	$\mathcal{U}$(-100, 100)	54.85	(21.94, 90.84)	-0.4189	28.97	(27.85, 30.79)	-1.473
q2	11.5	$\mathcal{U}$(1e-7, 50)	15.86	(5.989, 22.65)	-0.4416	1.161	(0.3452, 1.969)	-0.3639
q3*	0.4812	$\mathcal{U}$(-5, 1)	0.3981	(0.3359, 0.5484)	-0.6558	0.09093	(0.08613, 0.09628)	-1.829
q4	52.45	$\mathcal{U}$(-100, 100)	57.94	(49.6, 63.12)	-1.104	38.16	(37.65, 38.63)	-2.083
q5	14.97	$\mathcal{U}$(1e-7, 50)	12.1	(9.079, 16.57)	-0.7154	16.87	(16.17, 17.58)	-1.577
q6*	0.01414	$\mathcal{U}$(-7, 0)	0.01171	(0.009782, 0.01371)	-1.577	0.008563	(0.008114, 0.008981)	-2.178

Supplementary Table 14: Summary of results for parameters of Nygren I_{to} model. *Parameters were searched in $\log_{10}$ space based on the scale of their published values and are presented in linear space. The prior for these parameters is still in the original $\log_{10}$ space.

$$
\begin{aligned}
I_{\mathrm{to}} &= g_{\mathrm{to}} o_{\mathrm{a}}^3 o_{\mathrm{i}} \left(\mathrm{V} - \mathrm{E_K}\right) \\
\frac{\mathrm{d}\phi}{\mathrm{dt}} &= \frac{\phi_\infty - \phi}{\tau_\phi}, \qquad \text{for } \phi = o_{\mathrm{a}}, o_{\mathrm{i}} \\
\tau_\phi &= \left(\alpha_\phi + \beta_\phi\right)^{-1}, \qquad \text{for } \phi = o_{\mathrm{a}}, o_{\mathrm{i}}
\end{aligned}
$$

$$
o_{\mathrm{a}(\infty)} = \frac{1.0}{1.0 + e^{(\mathrm{V}+\mathbf{p_1})/-\mathbf{p_2}}}, \qquad \alpha_{o(\mathrm{a})} = \frac{\mathbf{p_3}}{e^{(\mathrm{V}+\mathbf{p_4})/-\mathbf{p_5}} + e^{(\mathrm{V}+\mathbf{p_6})/-\mathbf{p_7}}}, \qquad \beta_{o(\mathrm{a})} = \frac{\mathbf{p_3}}{\mathbf{p_8} + e^{(\mathrm{V}+\mathbf{p_9})/\mathbf{p_{10}}}}
$$

$$
o_{\mathrm{i}(\infty)} = \frac{1.0}{1.0 + e^{(\mathrm{V}+\mathbf{q_1})/\mathbf{q_2}}}, \qquad \alpha_{o(\mathrm{i})} = \frac{1.0}{\mathbf{q_3} + e^{(\mathrm{V}+\mathbf{q_4})/\mathbf{q_5}}}, \qquad \beta_{o(\mathrm{i})} = \frac{1.0}{\mathbf{q_6} + e^{(\mathrm{V}+\mathbf{q_7})/-\mathbf{q_8}}}
$$

Supplementary Table 15: Gating kinetics in Courtemanche model of I_{to} channel current (see Appendix in [2]).

Original/Unified					
Name	Published	Prior	Median	89% HDPI	$\log_{10}$RSD
p1	20.47	$\mathcal{U}$(-100, 100)	16.34	(7.558, 24.01)	-0.5041
p2	17.54	$\mathcal{U}$(1e-7, 50)	19.03	(14.78, 23.36)	-0.8263
p3*	0.65	$\mathcal{U}$(-3, 2)	0.06738	(0.002023, 0.259)	-0.3748
p4	10.0	$\mathcal{U}$(-100, 100)	28.32	(-0.545, 81.34)	-0.1259
p5	8.5	$\mathcal{U}$(1e-7, 50)	12.98	(0.01876, 41.94)	-0.09246
p6	-30.0	$\mathcal{U}$(-100, 100)	23.19	(4.345, 94.19)	-0.07312
p7	59.0	$\mathcal{U}$(1e-7, 100)	10.41	(0.008675, 37.07)	-0.05074
p8*	2.5	$\mathcal{U}$(-3, 2)	0.1598	(0.001051, 2.304)	0.04982
p9	82.0	$\mathcal{U}$(-100, 100)	62.09	(-8.713, 99.55)	-0.08063
p10	17.0	$\mathcal{U}$(1e-7, 50)	41.35	(21.91, 49.99)	-0.5362
q1	43.1	$\mathcal{U}$(-100, 100)	33.51	(30.65, 35.14)	-1.353
q2	5.3	$\mathcal{U}$(1e-7, 50)	6.981	(5.946, 8.025)	-0.8174
q3*	18.53	$\mathcal{U}$(-1, 4)	3.668	(0.1006, 14.48)	0.1733
q4	113.7	$\mathcal{U}$(0, 200)	125.8	(114.5, 135.2)	-1.178
q5	10.95	$\mathcal{U}$(1e-7, 50)	15.42	(13.43, 17.26)	-0.9771
q6*	35.56	$\mathcal{U}$(-1, 4)	37.67	(36.17, 41.82)	-1.898
q7	1.26	$\mathcal{U}$(-100, 100)	31.78	(27.76, 36.86)	-0.7325
q8	7.44	$\mathcal{U}$(1e-7, 50)	0.6272	(0.003919, 1.28)	0.1188

Supplementary Table 16: Summary of results for parameters of Courtemanche I_{to} model. Original and unified datasets are equivalent. *Parameters were searched in $\log_{10}$ space based on the scale of their published values and are presented in linear space. The prior for these parameters is still in the original $\log_{10}$ space.

Unified				
Name	Prior	Median	89% HDPI	$\log_{10}$RSD
p_1^*	$\mathcal{U}$(-7, 3)	0.00556	(0.005179, 0.006032)	-1.904
p_2	$\mathcal{U}$(1e-7, 0.4)	0.07096	(0.06763, 0.07326)	-1.586
p_3^*	$\mathcal{U}$(-7, 3)	0.1906	(0.1817, 0.1991)	-1.774
p_4	$\mathcal{U}$(1e-7, 0.4)	0.02528	(0.02459, 0.0261)	-1.724
p_5^*	$\mathcal{U}$(-7, 3)	0.1066	(0.1019, 0.1129)	-1.83
p_6	$\mathcal{U}$(1e-7, 0.4)	0.05923	(0.05696, 0.06139)	-1.57
p_7^*	$\mathcal{U}$(-7, 3)	0.0002949	(0.0002315, 0.000421)	-1.665
p_8	$\mathcal{U}$(1e-7, 0.4)	0.08746	(0.08027, 0.08944)	-1.478

Supplementary Table 17: Summary of results for parameters of standardised I_{to} model using unified dataset. *Parameters were searched in $\log_{10}$ space and are presented in linear space. The prior for these parameters is still in the original $\log_{10}$ space.

S4.4 I_{Kur}

$$
\begin{aligned}
I_{\mathrm{sus}} &= \bar{g}_{\mathrm{sus}} r_{\mathrm{sus}} s_{\mathrm{sus}} \left(\mathrm{V} - \mathrm{E_K}\right) \\
\frac{\mathrm{d}r_{\mathrm{sus}}}{\mathrm{dt}} &= \frac{\overline{r_{\mathrm{sus}}} - r_{\mathrm{sus}}}{\tau_{r_{\mathrm{sus}}}}, \qquad \frac{\mathrm{d}s_{\mathrm{sus}}}{\mathrm{dt}} = \frac{\overline{s_{\mathrm{sus}}} - s_{\mathrm{sus}}}{\tau_{s_{\mathrm{sus}}}} \\
\overline{r_{\mathrm{sus}}} &= \frac{1.0}{1.0 + e^{(\mathrm{V}+\mathbf{p_1})/-\mathbf{p_2}}}, \qquad \tau_{r_{\mathrm{sus}}} = 1000 \left(\frac{\mathbf{p_3}}{1.0 + e^{(\mathrm{V}+\mathbf{p_4})/\mathbf{p_5}}} + \mathbf{p_6} \right) \\
\overline{s_{\mathrm{sus}}} &= \frac{1.0 - \mathbf{q_3}}{1.0 + e^{(\mathrm{V}+\mathbf{q_1})/\mathbf{q_2}}} + \mathbf{q_3}, \qquad \tau_{s_{\mathrm{sus}}} = 1000 \left(\frac{\mathbf{q_4}}{1.0 + e^{(\mathrm{V}+\mathbf{q_5})/\mathbf{q_6}}} + \mathbf{q_7} \right)
\end{aligned}
$$

Supplementary Table 18: Gating kinetics in Nygren model of I_{sus} (I_{Kur}) channel current (see Table 8 in [1]). Time constants are multiplied by 1000 to convert from s to ms.

			Original			Unified		
Name	Published	Prior	Median	89% HDPI	$\log_{10}$RSD	Median	89% HDPI	$\log_{10}$RSD
p1	4.3	$\mathcal{U}$(-100, 100)	1.895	(1.821, 1.946)	-1.68	2.031	(1.996, 2.056)	-2.028
p2	8.0	$\mathcal{U}$(1e-7, 50)	4.54	(4.487, 4.564)	-2.226	4.305	(4.295, 4.325)	-2.61
p3*	0.009	$\mathcal{U}$(-5, 0)	0.004119	(0.004024, 0.004169)	-2.667	0.002799	(0.002775, 0.002824)	-3.017
p4	5.0	$\mathcal{U}$(-100, 100)	-13.6	(-14.2, -13.1)	-1.582	-19.02	(-19.31, -18.77)	-2.056
p5	12.0	$\mathcal{U}$(1e-7, 50)	7.566	(7.169, 7.953)	-1.496	5.109	(4.959, 5.271)	-1.709
p6*	0.0005	$\mathcal{U}$(-6, -1)	0.0007858	(0.0007327, 0.0008531)	-2.147	0.0008455	(0.0008235, 0.0008719)	-2.58
q1	20.0	$\mathcal{U}$(-100, 100)	21.14	(8.065, 51.78)	-0.1842	21.14	(8.065, 51.78)	-0.1842
q2	10.0	$\mathcal{U}$(1e-7, 50)	12.96	(0.8947, 27.71)	-0.2282	12.96	(0.8947, 27.71)	-0.2282
q3	0.6	$\mathcal{U}$(0, 1)	0.121	(0.0001271, 0.268)	-0.1557	0.121	(0.0001271, 0.268)	-0.1557
q4*	0.047	$\mathcal{U}$(-4, 1)	0.06416	(0.01542, 0.133)	-0.6109	0.06416	(0.01542, 0.133)	-0.6109
q5	60.0	$\mathcal{U}$(-100, 100)	43.27	(-52.21, 99.65)	0.2045	43.27	(-52.21, 99.65)	0.2045
q6	10.0	$\mathcal{U}$(1e-7, 50)	27.02	(1.936, 45.79)	-0.238	27.02	(1.936, 45.79)	-0.238
q7*	0.3	$\mathcal{U}$(-3, 2)	0.286	(0.2161, 0.3196)	-0.221	0.286	(0.2161, 0.3196)	-0.221

Supplementary Table 19: Summary of results for parameters of Nygren I_{Kur} model. Only activation parameters (p) were recalibrated to unified dataset. *Parameters were searched in $\log_{10}$ space based on the scale of their published values and are presented in linear space. The prior for these parameters is still in the original $\log_{10}$ space.

$$I_{\mathrm{Kur}} = g_{\mathrm{Kur}} \left(1.0 + \frac{\mathbf{r_1}}{1.0 + e^{(V+\mathbf{r_2})/-\mathbf{r_3}}}\right) u_{\mathrm{a}}^3 u_{\mathrm{i}} \left(\mathrm{V} - \mathrm{E_K}\right)$$

$$\frac{\mathrm{d}u_{\mathrm{a}}}{\mathrm{dt}} = \frac{u_{\mathrm{a}(\infty)} - u_{\mathrm{a}}}{\tau_{u(\mathrm{a})}}, \qquad \frac{\mathrm{d}u_{\mathrm{i}}}{\mathrm{dt}} = \frac{u_{\mathrm{i}(\infty)} - u_{\mathrm{i}}}{\tau_{u(\mathrm{i})}}$$

$$u_{\mathrm{a}(\infty)} = \frac{1.0}{1.0 + e^{(\mathrm{V}+\mathbf{p_1})/-\mathbf{p_2}}}, \qquad \alpha_{u(\mathrm{a})} = \frac{\mathbf{p_3}}{e^{(\mathrm{V}+\mathbf{p_4})/-\mathbf{p_5}} + e^{(\mathrm{V}+\mathbf{p_6})/-\mathbf{p_7}}}, \qquad \beta_{u(\mathrm{a})} = \frac{\mathbf{p_3}}{\mathbf{p_8} + e^{(\mathrm{V}+\mathbf{p_9})/\mathbf{p_10}}}$$

$$u_{\mathrm{i}(\infty)} = \frac{1.0}{1.0 + e^{(\mathrm{V}+\mathbf{q_1})/\mathbf{q_2}}}, \qquad \alpha_{u(\mathrm{i})} = \frac{1.0}{\mathbf{q_3} + e^{(\mathrm{V}+\mathbf{q_4})/-\mathbf{q_5}}}, \qquad \beta_{u(\mathrm{i})} = e^{(\mathrm{V}+\mathbf{q_6})/\mathbf{q_7}}$$

$$\tau_\phi = \left(\alpha_\phi + \beta_\phi\right)^{-1}, \qquad \text{for } \phi = u_{\mathrm{a}}, u_{\mathrm{i}}$$

Supplementary Table 20: Gating kinetics in Courtemanche model of I_{Kur} channel current (see Appendix in [2]).

			Original			Unified		
Name	Published	Prior	Median	89% HDPI	$\log_{10}$RSD	Median	89% HDPI	$\log_{10}$RSD
p1	30.3	$\mathcal{U}$(-100, 100)	22.15	(20.09, 24.22)	-1.213	22.15	(20.09, 24.22)	-1.213
p2	9.6	$\mathcal{U}$(1e-7, 50)	17.41	(13.63, 21.67)	-0.8446	17.41	(13.63, 21.67)	-0.8446
p3*	0.65	$\mathcal{U}$(-3, 2)	0.04404	(0.00102, 0.1094)	-0.4886	0.04404	(0.00102, 0.1094)	-0.4886
p4	10.0	$\mathcal{U}$(-100, 100)	32.29	(-4.553, 88.19)	-0.1005	32.29	(-4.553, 88.19)	-0.1005
p5	8.5	$\mathcal{U}$(1e-7, 50)	16.13	(3.228, 37.54)	-0.2495	16.13	(3.228, 37.54)	-0.2495
p6	-30.0	$\mathcal{U}$(-100, 100)	32.82	(-2.739, 87.54)	-0.1255	32.82	(-2.739, 87.54)	-0.1255
p7	59.0	$\mathcal{U}$(1e-7, 50)	16.26	(1.388, 32.48)	-0.2607	16.26	(1.388, 32.48)	-0.2607
p8*	2.5	$\mathcal{U}$(-3, 2)	0.06393	(0.001001, 0.5319)	-0.1877	0.06393	(0.001001, 0.5319)	-0.1877
p9	82.0	$\mathcal{U}$(-100, 100)	47.77	(-22.92, 99.54)	0.04513	47.77	(-22.92, 99.54)	0.04513
p10	17.0	$\mathcal{U}$(1e-7, 50)	41.82	(29.25, 49.78)	-0.6841	41.82	(29.25, 49.78)	-0.6841
r1*	10.0	$\mathcal{U}$(0, 2)	8.721	(1.004, 61.18)	-0.2278	8.721	(1.004, 61.18)	-0.2278
r2	-15.0	$\mathcal{U}$(-100, 100)	-6.353	(-84.56, 87.51)	1.22	-6.353	(-84.56, 87.51)	1.22
r3	13.0	$\mathcal{U}$(1e-7, 50)	25.3	(0.04052, 44.01)	-0.231	25.3	(0.04052, 44.01)	-0.231
q1	-99.45	$\mathcal{U}$(-200, 200)	-69.32	(-144.5, -0.2399)	-0.1766	54.84	(54.83, 54.85)	-3.809
q2	27.48	$\mathcal{U}$(1e-7, 50)	34.61	(15.72, 49.44)	-0.4477	39.95	(39.94, 39.96)	-3.968
q3*	21.0	$\mathcal{U}$(-1, 4)	1941.0	(0.1185, 5866.0)	-0.319	674.0	(674.0, 674.0)	-5.458
q4	-185.0	$\mathcal{U}$(-200, 200)	-118.8	(-199.4, -34.61)	-0.2815	74.15	(72.39, 81.09)	-1.373
q5	28.0	$\mathcal{U}$(1e-7, 50)	26.47	(0.6525, 43.54)	-0.2649	3.708	(2.084, 4.118)	-0.6464
q6	-158.0	$\mathcal{U}$(-200, 0)	-172.1	(-199.5, -133.3)	-0.8272	-151.0	(-199.9, -95.79)	-0.6078
q7	16.0	$\mathcal{U}$(1e-7, 50)	23.66	(14.8, 28.97)	-0.5748	1.984	(0.03531, 4.561)	-0.1569

Supplementary Table 21: Summary of results for parameters of Courtemanche I_{Kur}. Only inactivation parameters (q) were recalibrated to unified dataset. *Parameters were searched in $\log_{10}$ space based on the scale of their published values and are presented in linear space. The prior for these parameters is still in the original $\log_{10}$ space.

Unified				
Name	Prior	Median	89% HDPI	$\log_{10}$RSD
p_1^*	$\mathcal{U}$(-7, 3)	0.0558	(0.0433, 0.06443)	-1.367
p_2	$\mathcal{U}$(1e-7, 0.4)	0.1464	(0.1281, 0.1683)	-1.043
p_3^*	$\mathcal{U}$(-7, 3)	0.1188	(0.1096, 0.1279)	-1.634
p_4	$\mathcal{U}$(1e-7, 0.4)	0.02021	(0.01904, 0.02213)	-1.306
p_5^*	$\mathcal{U}$(-7, 3)	0.004436	(0.004311, 0.004566)	-2.485
p_6	$\mathcal{U}$(1e-7, 0.4)	0.001568	(0.001221, 0.001863)	-0.8671
p_7^*	$\mathcal{U}$(-7, 3)	1.424e-07	(1.005e-07, 3.607e-07)	-1.417
p_8	$\mathcal{U}$(1e-7, 0.4)	0.02784	(0.02351, 0.03098)	-1.079

Supplementary Table 22: Summary of results for parameters of standardised I_{Kur} model using unified dataset. *Parameters were searched in $\log_{10}$ space and are presented in linear space. The prior for these parameters is still in the original $\log_{10}$ space.

References

[1] Nygren A, Fiset C, Firek L, Clark JW, Lindblad DS, Clark RB, Giles WR. 1998 Mathematical model of an adult human atrial cell: the role of K+ currents in repolarization. *Circulation Research* **82**, 63–81. (doi:10.1161/01.res.82.1.63)

[2] Courtemanche M, Ramirez RJ, Nattel S. 1998 Ionic mechanisms underlying human atrial action potential properties: insights from a mathematical model. *The American Journal of Physiology* **275**, H301–321. (doi:10.1152/ajpheart.1998.275.1.H301)

[3] Sakakibara Y, Wasserstrom JA, Furukawa T, Jia H, Arentzen CE, Hartz RS, Singer DH. 1992 Characterization of the sodium current in single human atrial myocytes. *Circulation Research* **71**, 535–546. (doi:10.1161/01.res.71.3.535)

[4] Schneider M, Proebstle T, Hombach V, Hannekum A, Rüdel R. 1994 Characterization of the sodium currents in isolated human cardiocytes. *Pflugers Archiv: European Journal of Physiology* **428**, 84–90. (doi:10.1007/bf00374755)

[5] Mewes T, Ravens U. 1994 L-type Calcium Currents of Human Myocytes from Ventricle of Non-failing and Failing Hearts and from Atrium. *Journal of Molecular and Cellular Cardiology* **26**, 1307–1320. (doi:10.1006/jmcc.1994.1149)

[6] Li GR, Nattel S. 1997 Properties of human atrial ICa at physiological temperatures and relevance to action potential. *The American Journal of Physiology* **272**, H227–235. (doi: 10.1152/ajpheart.1997.272.1.H227)

[7] Sun H, Leblanc N, Nattel S. 1997 Mechanisms of inactivation of L-type calcium channels in human atrial myocytes. *The American Journal of Physiology* **272**, H1625–1635. (doi: 10.1152/ajpheart.1997.272.4.H1625)

[8] Shibata EF, Drury T, Refsum H, Aldrete V, Giles W. 1989 Contributions of a transient outward current to repolarization in human atrium. *American Journal of Physiology-Heart and Circulatory Physiology* **257**, H1773–H1781. (doi:10.1152/ajpheart.1989.257.6.H1773)

[9] Wang Z, Fermini B, Nattel S. 1993 Sustained depolarization-induced outward current in human atrial myocytes. Evidence for a novel delayed rectifier K+ current similar to Kv1.5 cloned channel currents. *Circulation Research* **73**, 1061–1076. (doi:10.1161/01.res.73.6.1061)

[10] Firek L, Giles WR. 1995 Outward currents underlying repolarization in human atrial myocytes. *Cardiovascular Research* **30**, 31–38.

[11] ten Tusscher KHWJ, Noble D, Noble PJ, Panfilov AV. 2004 A model for human ventricular tissue. *American Journal of Physiology. Heart and Circulatory Physiology* **286**, H1573–1589. (doi: 10.1152/ajpheart.00794.2003)

[12] Toni T, Welch D, Strelkowa N, Ipsen A, Stumpf MPH. 2009 Approximate Bayesian computation scheme for parameter inference and model selection in dynamical systems. *Journal of the Royal Society, Interface* **6**, 187–202. (doi:10.1098/rsif.2008.0172)

[13] Mirams GR, Pathmanathan P, Gray RA, Challenor P, Clayton RH. 2016 Uncertainty and variability in computational and mathematical models of cardiac physiology. *The Journal of Physiology* **594**, 6833–6847. (doi:10.1113/JP271671)

[14] Beattie KA, Hill AP, Bardenet R, Cui Y, Vandenberg JI, Gavaghan DJ, de Boer TP, Mirams GR. 2018 Sinusoidal voltage protocols for rapid characterisation of ion channel kinetics. *The Journal of Physiology* **596**, 1813–1828. (doi:10.1113/JP275733)
